# Supplementary material for: Stretchable and negative-Poisson-ratio porous metamaterials
Source: Nat Commun. 2024 Jan 9;15:392. doi: 10.1038/s41467-024-44707-3 (PMC10776607; doi:10.1038/s41467-024-44707-3)
Supplement: Supplementary file 1 — Supplementary Information [file 41467_2024_44707_MOESM1_ESM.pdf]

Supplementary Information

Stretchable and negative-Poisson-ratio porous metamaterials

Xiaoyu Zhang[1], Qi Sun[1], Xing Liang[1], Puzhong Gu[1], Zhenyu Hu[1], Xiao Yang[1], Muxiang Liu[1], Zejun Sun[1], Jia Huang[1], Guangming Wu[2], Guoqing Zu*[,1]

[1]Interdisciplinary Materials Research Center, Department of Polymeric Materials, School of Materials Science and Engineering, Tongji University, Shanghai, 201804, P. R. China

[2]Shanghai Key Laboratory of Special Artificial Microstructure Materials and Technology, School of Physics Science and Engineering, Tongji University, Shanghai 200092, P. R. China

*Corresponding author: guoqingzu@tongji.edu.cn

This file includes:

- Captions for Supplementary Movies (1-10)
- Supplementary Figures (1-49)
- Supplementary Tables (1-6)
- Supplementary Notes (1-6)
- Supplementary References (1-66)

Other Supplementary Materials for this manuscript include:

Supplementary Movies (1-10)

Movie list:

Supplementary Movie 1. A tensile test in *x* direction on rGO/PPy/PUF1-UHP, showing an elongation at break of 810%.

Supplementary Movie 2. A tensile test in *x* direction on rGO/PPy/PUF2-UHP, showing an elongation at break of 1250%.

Supplementary Movie 3. A stretching-releasing test in *x* direction on rGO/PPy/PUF1-UHP with 700% tensile strain.

Supplementary Movie 4. A stretching-releasing test in *x* direction on rGO/PPy/PUF2-UHP with 800% tensile strain.

Supplementary Movie 5. A tensile test in *x* direction on rGO/PPy/PUF1-BHP with 300% tensile strain.

Supplementary Movie 6. A stretching-releasing test in *y* direction on rGO/PPy/PUF1-BHP with 300% tensile strain.

Supplementary Movie 7. A tensile test in *y* direction on rGO/PPy/PUF1-THP, showing an elongation at break of 340%.

Supplementary Movie 8. FEA simulation of the structure variation of the elastomer during uniaxial hot pressing.

Supplementary Movie 9. FEA simulation of the structure variation of the elastomer during biaxial hot pressing.

Supplementary Movie 10. Monitoring the large tensile strain (0-300%) of a chest developer during exercise by using the strain sensor based on rGO/PPy/PUF1-UHP.

Supplementary Figures

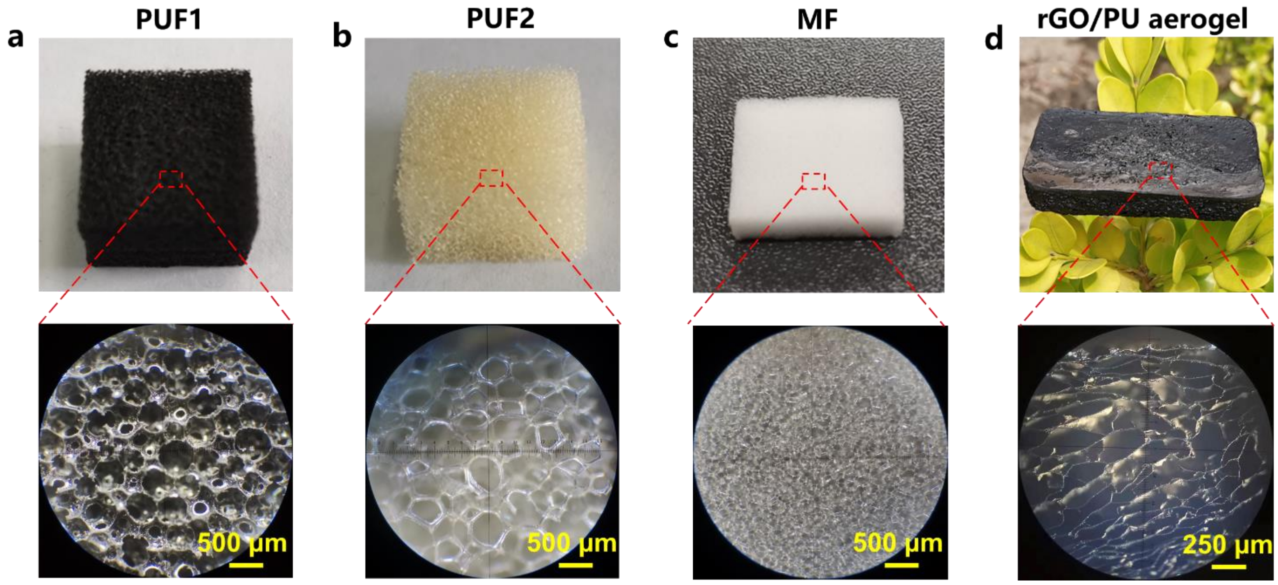

Supplementary Fig. 1. Photographs and optical microscope images of **a** PUF1, **b** PUF2, **c** MF, and **d** the rGO/PU aerogel. Two PUFs with different bulk densities (79 mg cm^{-3} for PUF1 and 18 mg cm^{-3} for PUF2) were used in this work.

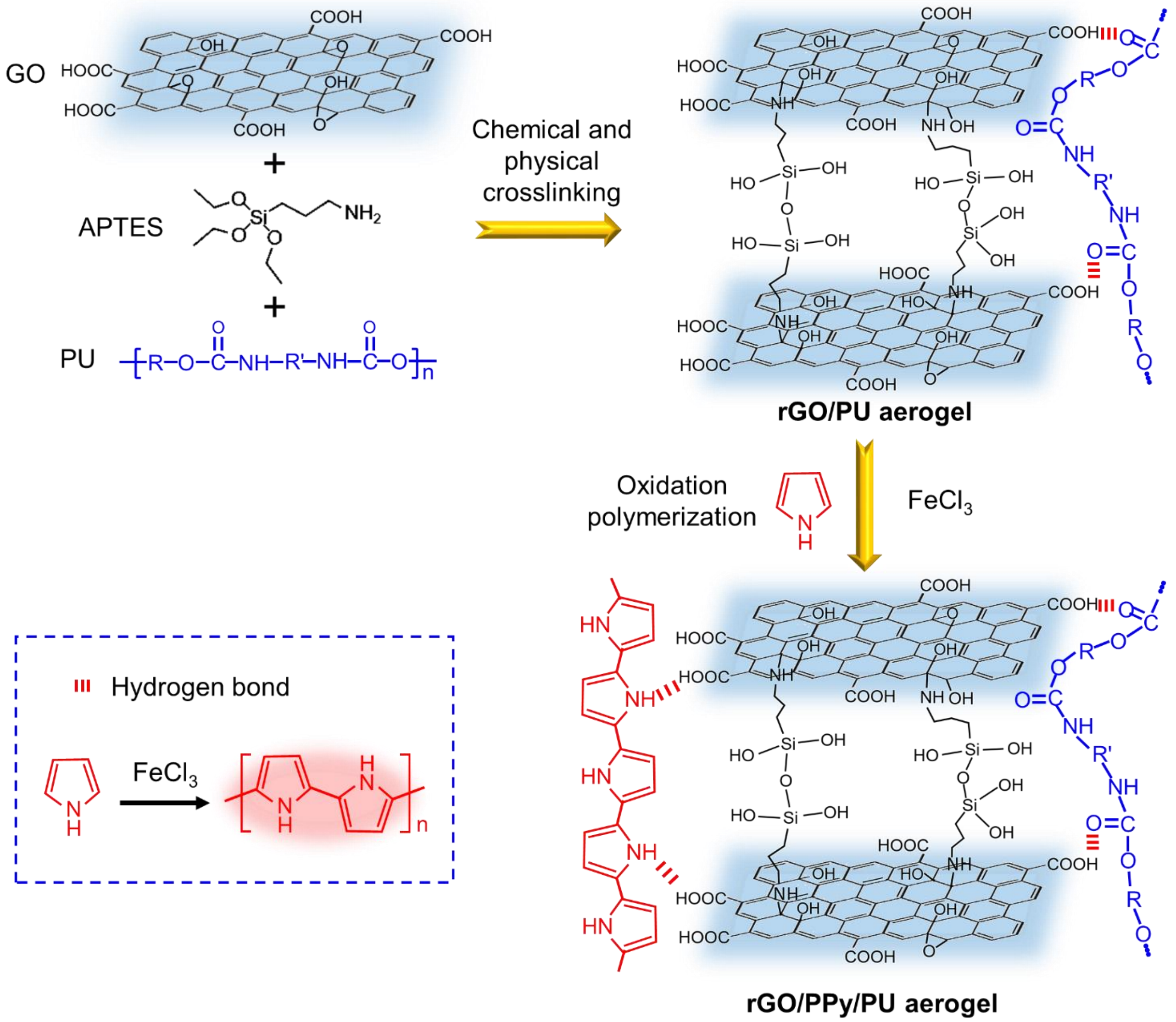

Supplementary Fig. 2. Schematic of the crosslinking and molecular-scaled microstructure variation during the preparation of the rGO/PPy/PU aerogel.

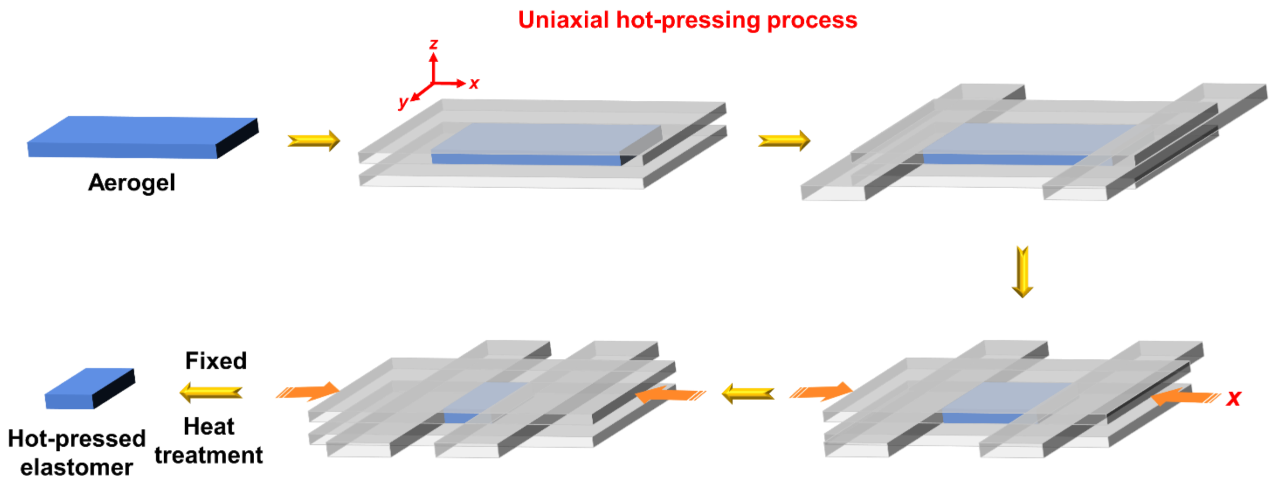

Supplementary Fig. 3. Schematic of the uniaxial hot-pressing process of the elastomer.

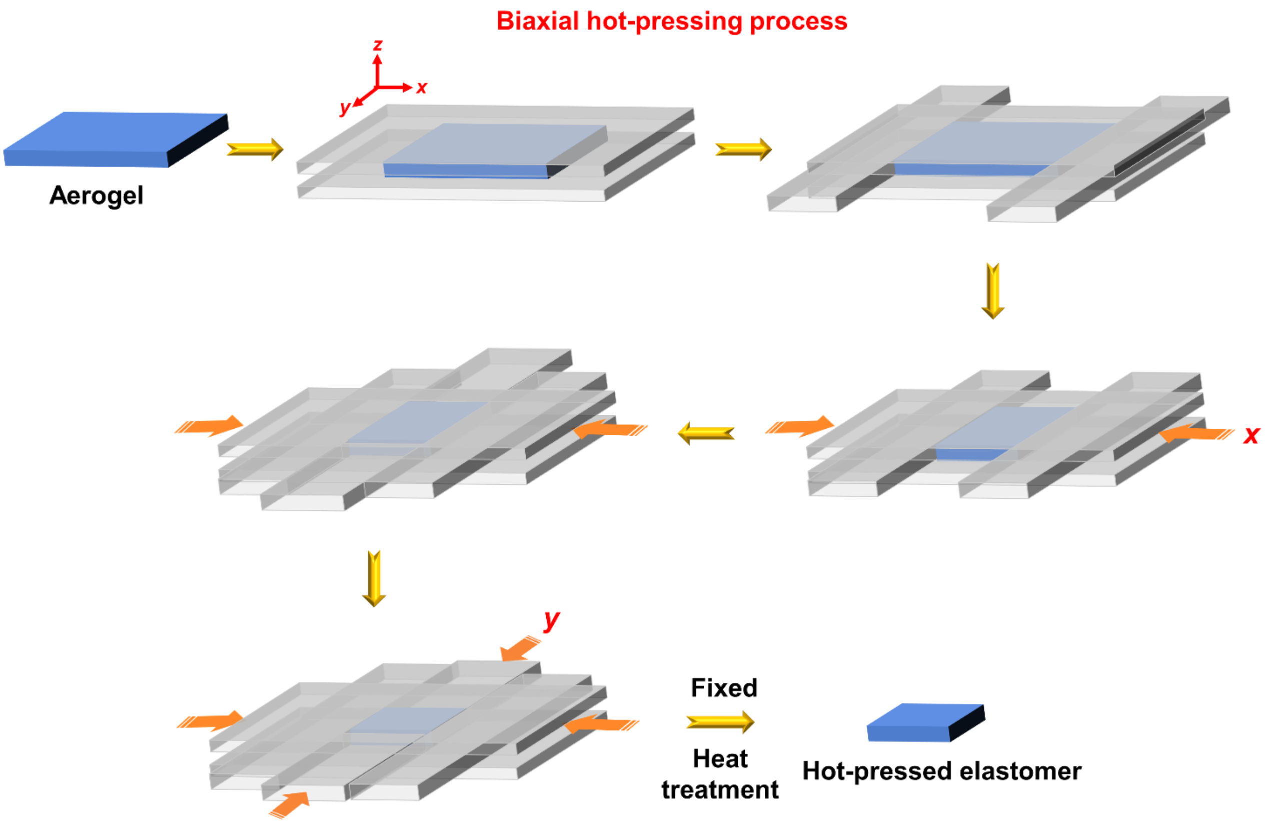

Supplementary Fig. 4. Schematic of the biaxial hot-pressing process of the elastomer.

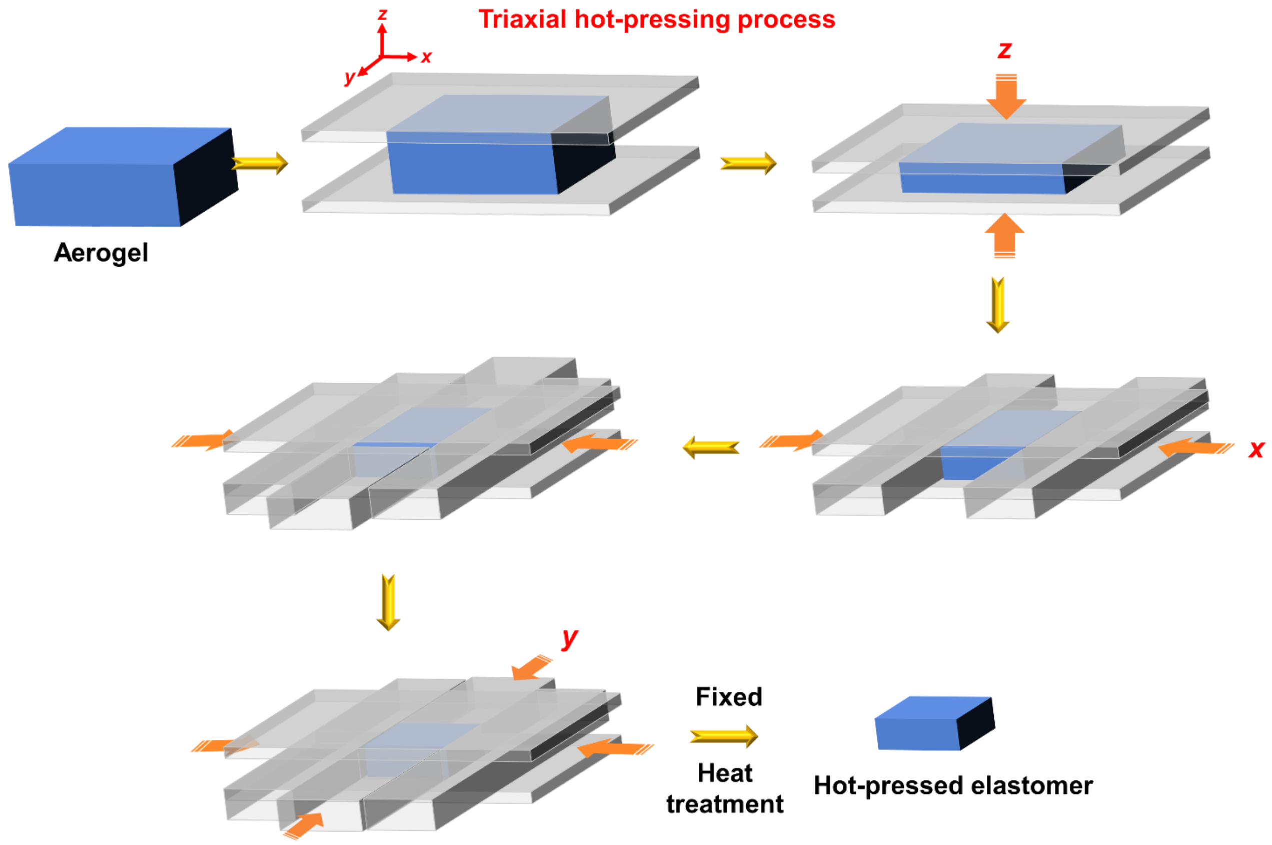

Supplementary Fig. 5. Schematic of the triaxial hot-pressing process of the elastomer.

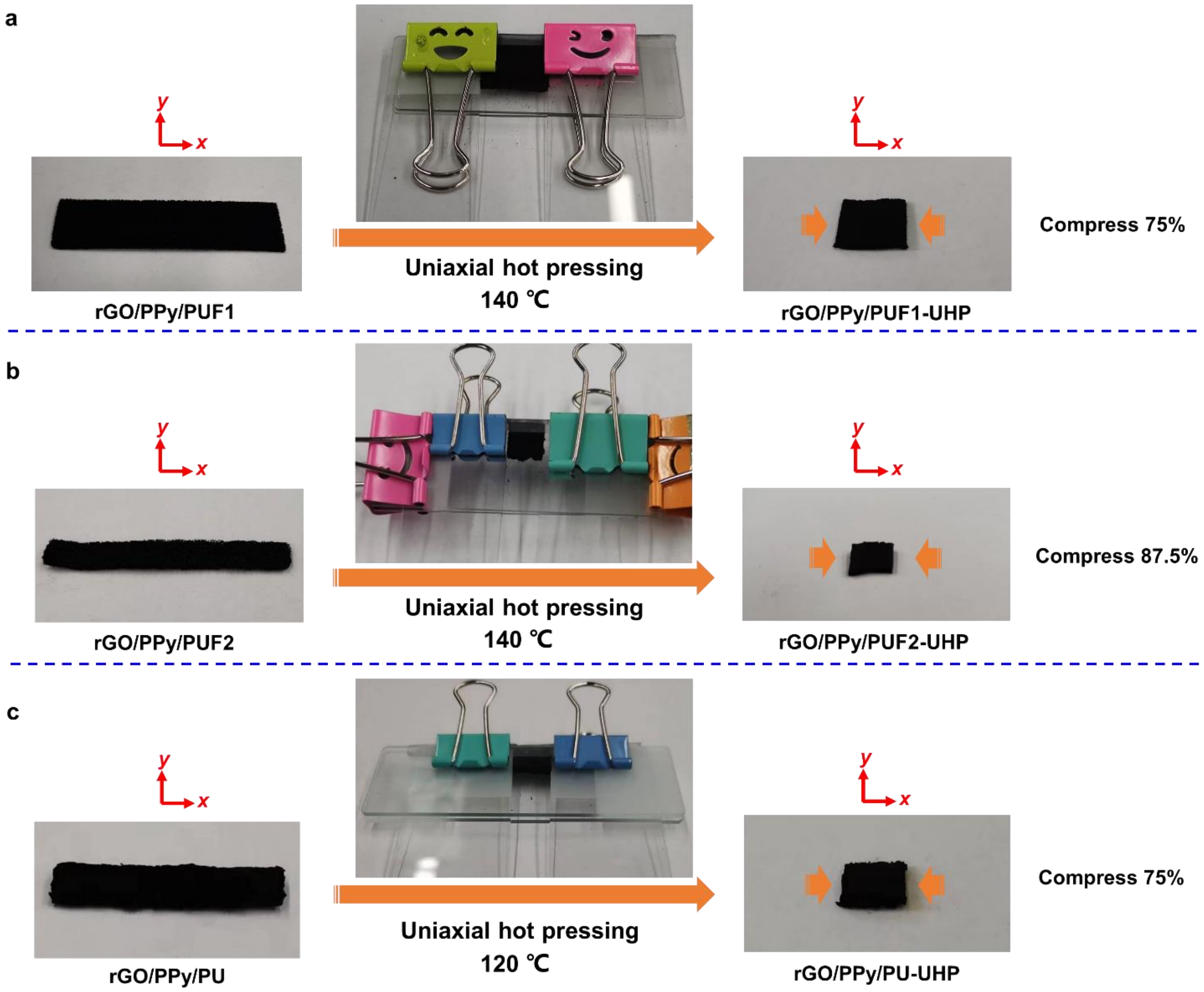

Supplementary Fig. 6. Photographs of the rGO/polymer elastomers before and after uniaxial hot pressing. **a** rGO/PPy/PUF1-UHP. **b** rGO/PPy/PUF2-UHP. **c** rGO/PPy/PU-UHP.

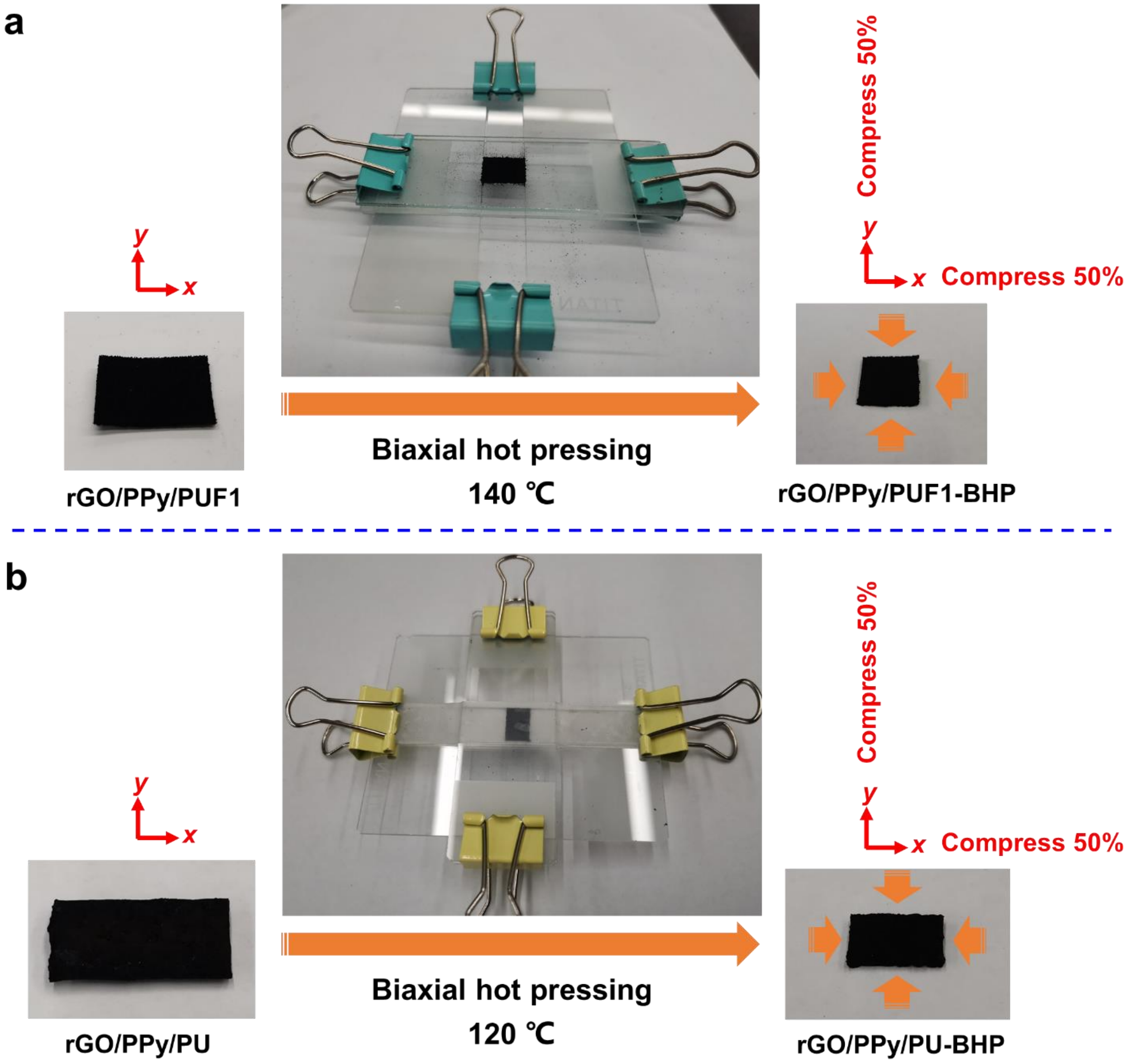

Supplementary Fig. 7. Photographs of the rGO/polymer elastomers before and after biaxial hot pressing. **a** rGO/PPy/PUF1-BHP. **b** rGO/PPy/PU-BHP.

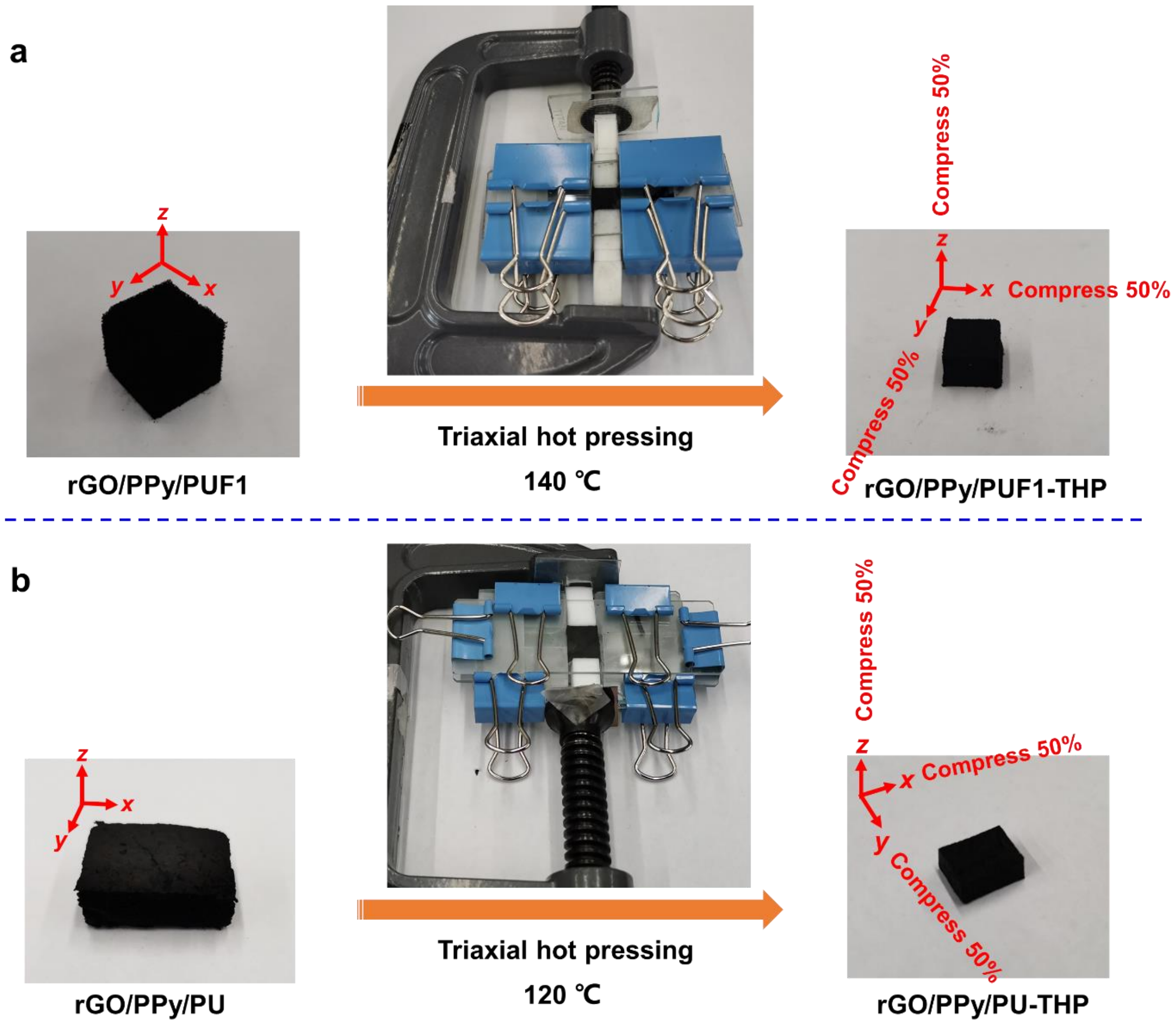

Supplementary Fig. 8. Photographs of the rGO/polymer elastomers before and after triaxial hot pressing. **a** rGO/PPy/PUF1-THP. **b** rGO/PPy/PU-THP.

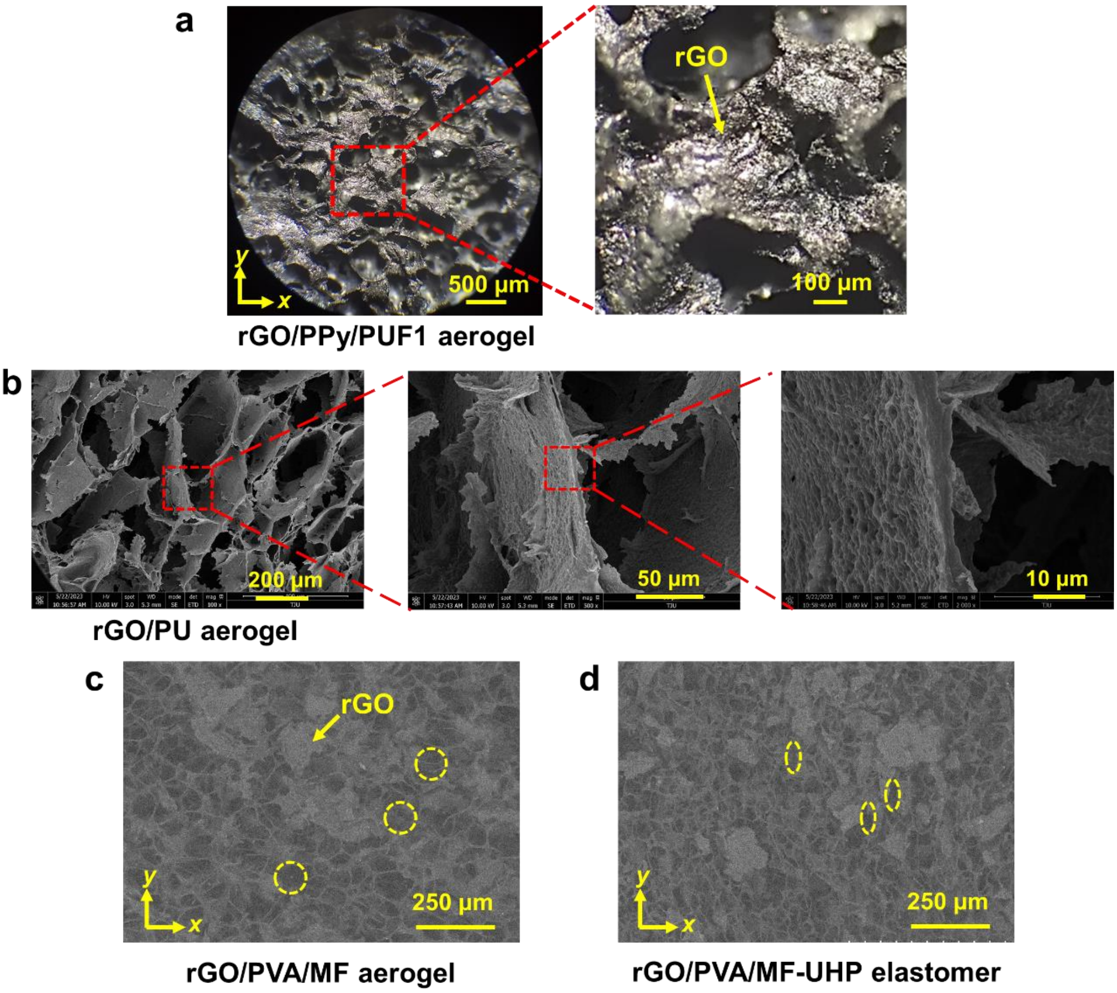

Supplementary Fig. 9. Morphologies of typical pristine rGO/polymer aerogels and hot-pressed elastomers. **a** Optical microscope image of the rGO/PPy/PUF1 aerogel without hot pressing. **b** SEM images of the rGO/PU aerogel. SEM images of the **c** pristine rGO/PVA/MF aerogel and **d** uniaxially hot-pressed rGO/PVA/MF-UHP elastomer. The uniaxially hot-pressed rGO/PVA/MF-UHP exhibited compressed and folded porous structures with smaller pores compared with those of pristine aerogels.

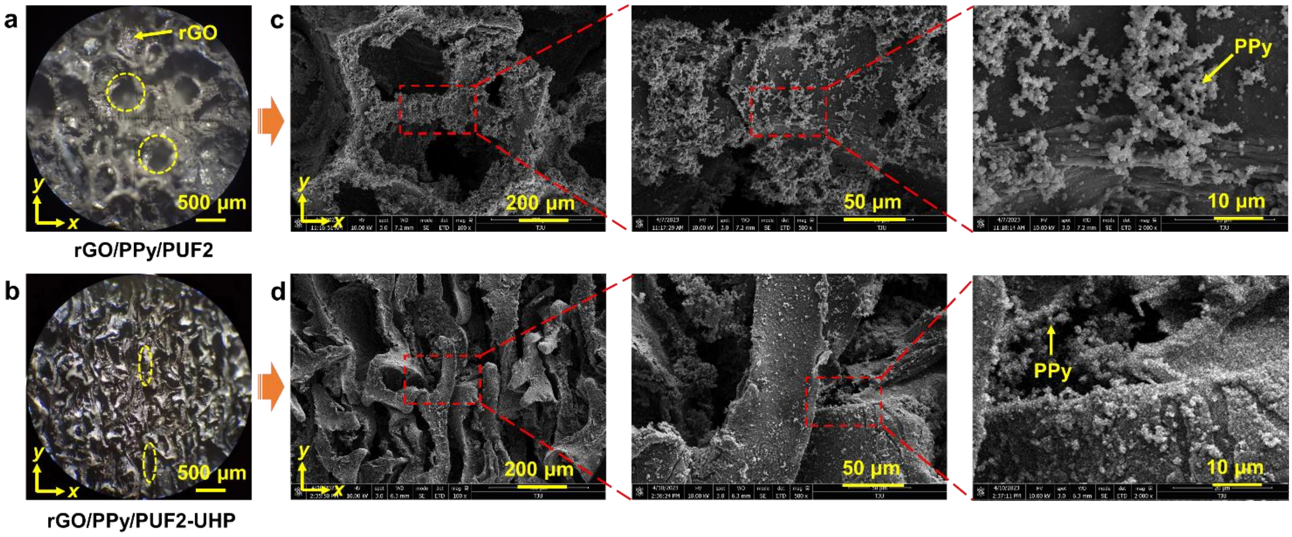

Supplementary Fig. 10. Morphologies of rGO/PPy/PUF2-UHP before and after uniaxial hot pressing. **a,b** optical microscope images. **c,d** SEM images. The rGO nanosheets and PPy particles were observed in the aerogels rGO/PPy/PUF2 and rGO/PPy/PUF2-UHP. The uniaxially hot-pressed elastomer rGO/PPy/PUF2-UHP exhibited compressed and folded porous structures with smaller pores compared with those of pristine rGO/PPy/PUF2.

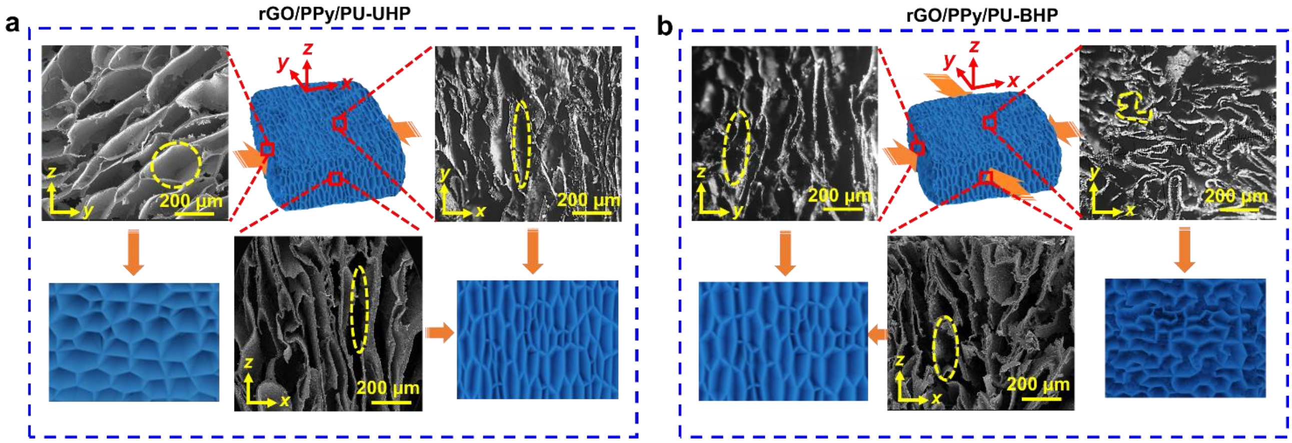

Supplementary Fig. 11. SEM images of the uniaxially and biaxially hot-pressed rGO/PU-based elastomers. **a** SEM images of the uniaxially hot-pressed rGO/PPy/PU-UHP in the *xy*, *xz*, and *yz* planes. **b** SEM images of the biaxially hot-pressed rGO/PPy/PU-BHP in the *xy*, *xz*, and *yz* planes.

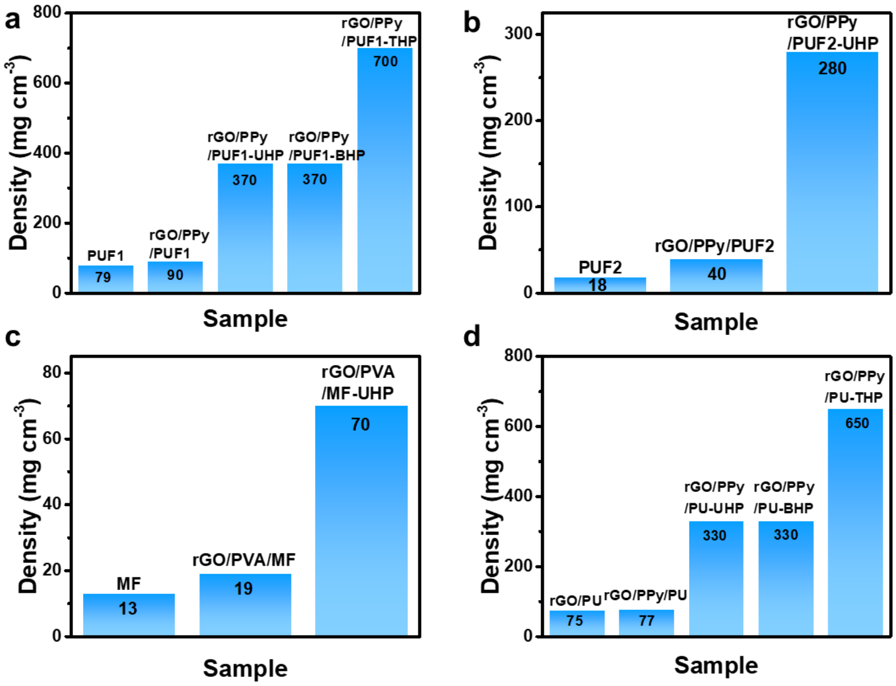

Supplementary Fig. 12. Bulk densities of typical porous materials. **a** Bulk densities of PUF1, rGO/PPy/PUF1, and hot-pressed rGO/PPy/PUF composite materials. **b** Bulk densities of PUF2, rGO/PPy/PUF2, and rGO/PPy/PUF2-UHP. **c** Bulk densities of MF, rGO/PVA/MF, and rGO/PVA/MF-UHP. **d** Bulk densities of rGO/PU, rGO/PPy/PU, and hot-pressed rGO/PPy/PU composite materials.

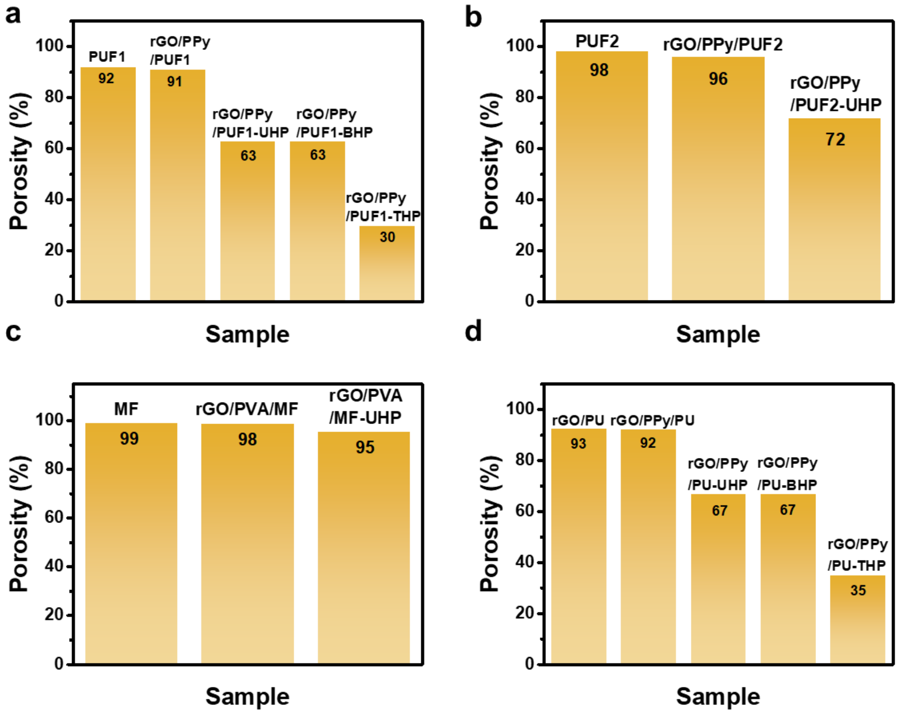

Supplementary Fig. 13. Porosities of typical porous materials. **a** Porosities of PUF1, rGO/PPy/PUF1, and hot-pressed rGO/PPy/PUF composite materials. **b** Porosities of PUF2, rGO/PPy/PUF2, and rGO/PPy/PUF2-UHP. **c** Porosities of MF, rGO/PVA/MF, and rGO/PVA/MF-UHP. **d** Porosities of rGO/PU, rGO/PPy/PU, and hot-pressed rGO/PPy/PU composite materials.

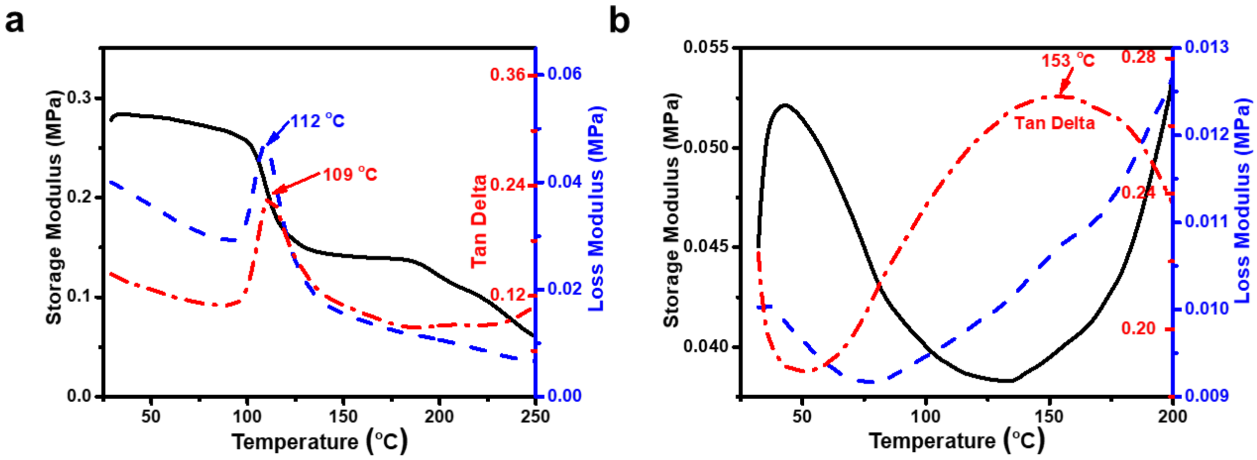

Supplementary Fig. 14. Storage modulus, loss modulus, and tan delta of **a** rGO/PPy/PUF1 and **b** rGO/PPy/PU. For rGO/PPy/PUF1, the peak of tan delta is located at approximately 110 °C. Accordingly, its storage modulus decreases obviously at around 110 °C, indicating that a softening process occurs at approximately 110 °C. For rGO/PPy/PU, the broad peak of tan delta is located at approximately 70-180 °C. Meanwhile, the storage modulus of rGO/PPy/PU decreases with the increase of temperature in the range of 50-130 °C. These results confirm that the rGO/polymer composite materials will undergo a softening process during hot pressing at 140 or 120 °C. Source data are provided as a Source Data file.

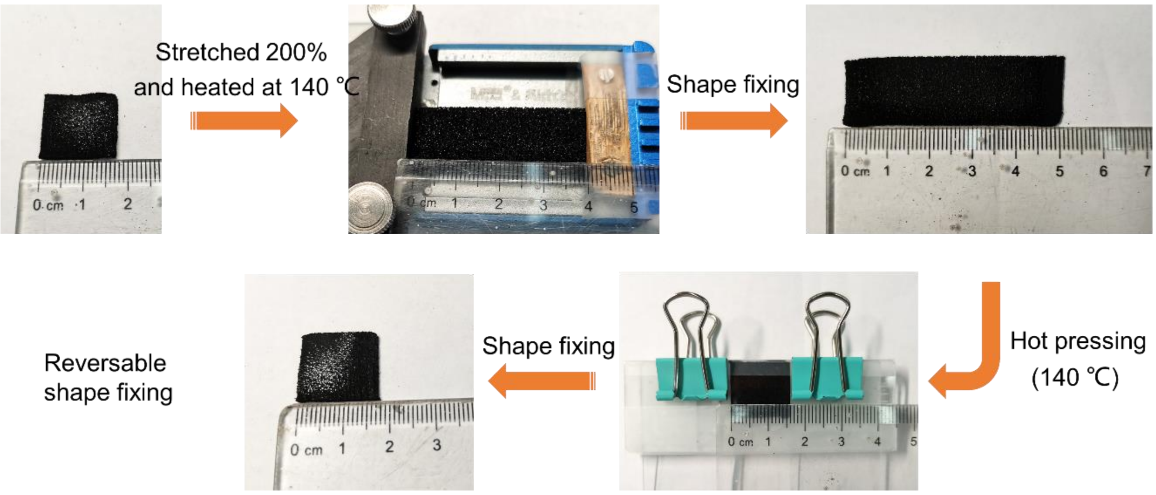

Supplementary Fig. 15. Reversable shape fixing process of rGO/PPy/PUF1-UHP. When rGO/PPy/PUF1-UHP is stretched 200% and heated at 140 °C for 5 h under vacuum condition, its shape can be fixed without springing back after cooling down to room temperature. The resultant material can be hot pressed (140 °C for 5 h under vacuum condition) to its initial size of rGO/PPy/PUF1-UHP. This confirms the reversable shape fixing of the rGO/polymer composite materials.

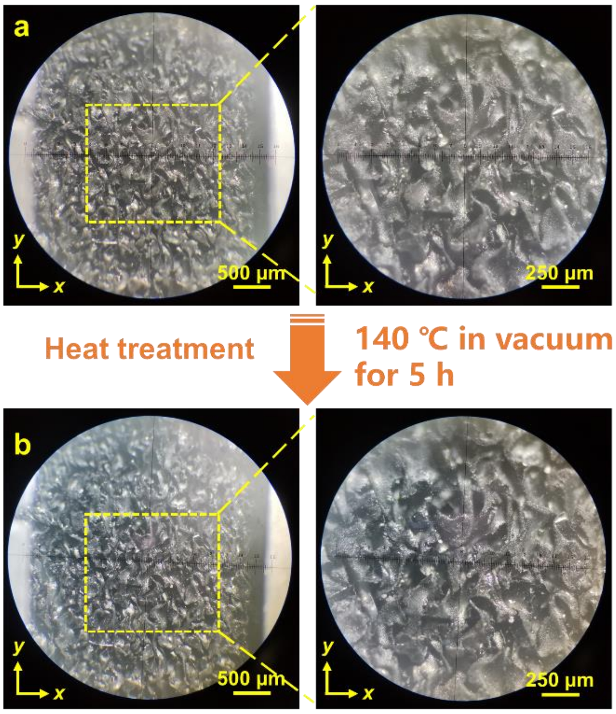

Supplementary Fig. 16. Optical microscope images of a) rGO/PPy/PUF1-UP and b) rGO/PPy/PUF1-UHP.

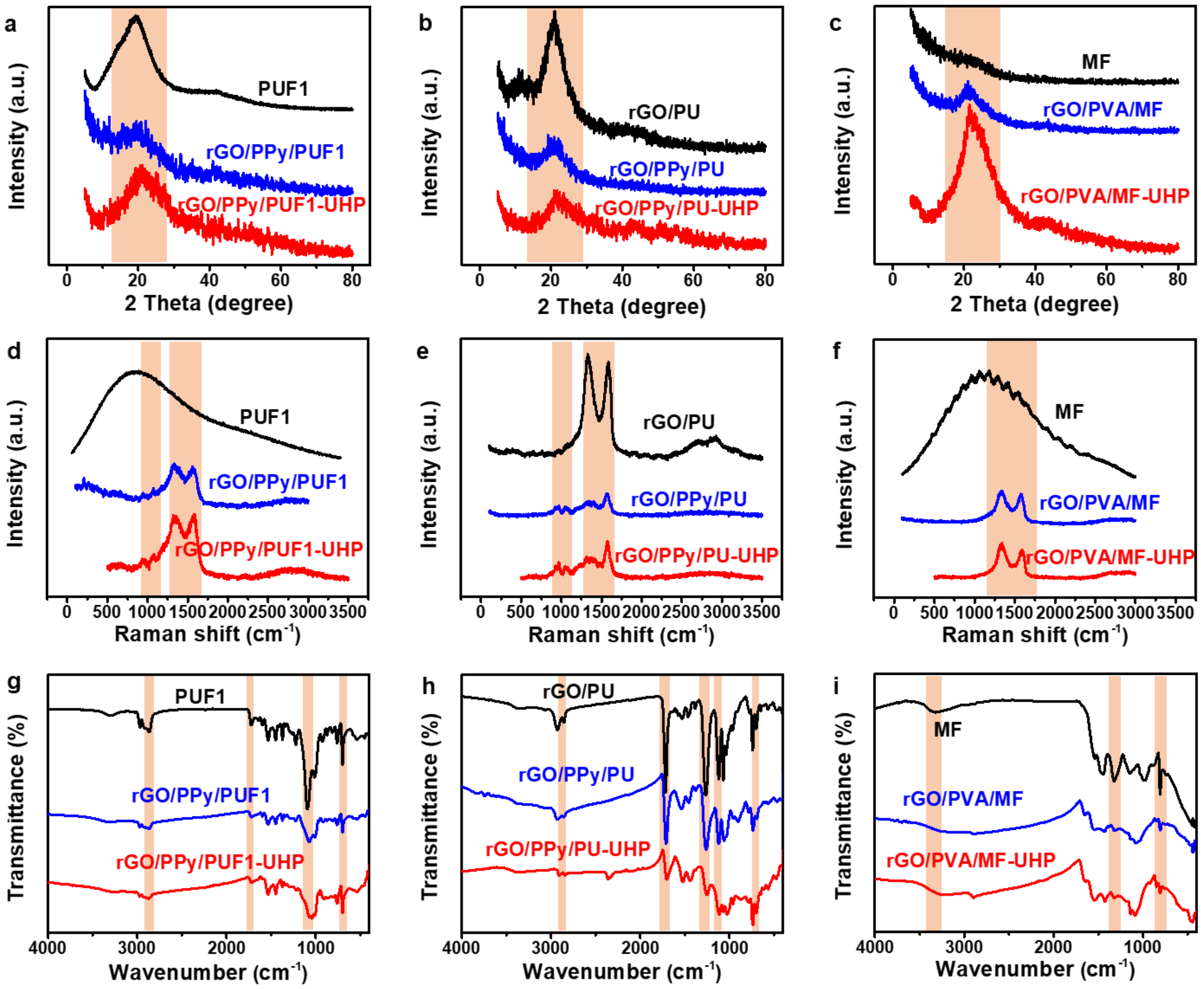

Supplementary Fig. 17. Chemical structures of typical pristine rGO/polymer aerogels and the hot-pressed elastomers. **a-c** XRD patterns of the polymers, aerogels, and hot-pressed elastomers. **d-f** Raman spectra of the polymers, aerogels, and hot-pressed elastomers. **g-i** FTIR spectra of the polymers, aerogels, and hot-pressed elastomers. Source data are provided as a Source Data file.

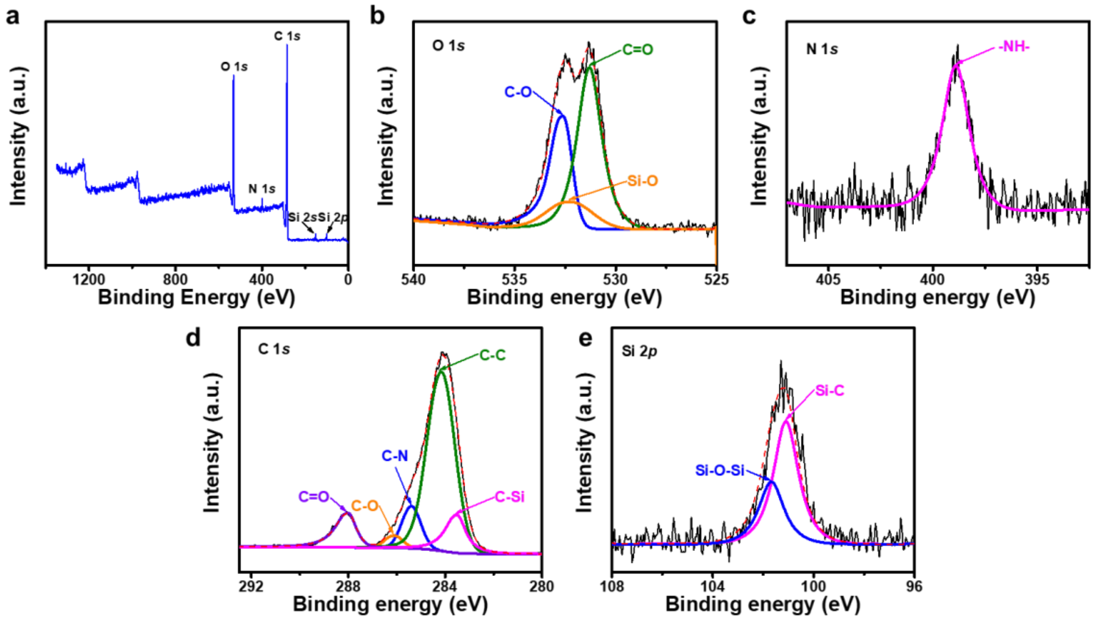

Supplementary Fig. 18. **a** XPS spectrum of the APTES-crosslinked rGO/PU aerogel. **b** XPS O 1*s*, **c** N 1*s*, **d** C 1*s*, and **e** Si 2*p* spectra of the APTES-crosslinked rGO/PU aerogel. Source data are provided as a Source Data file.

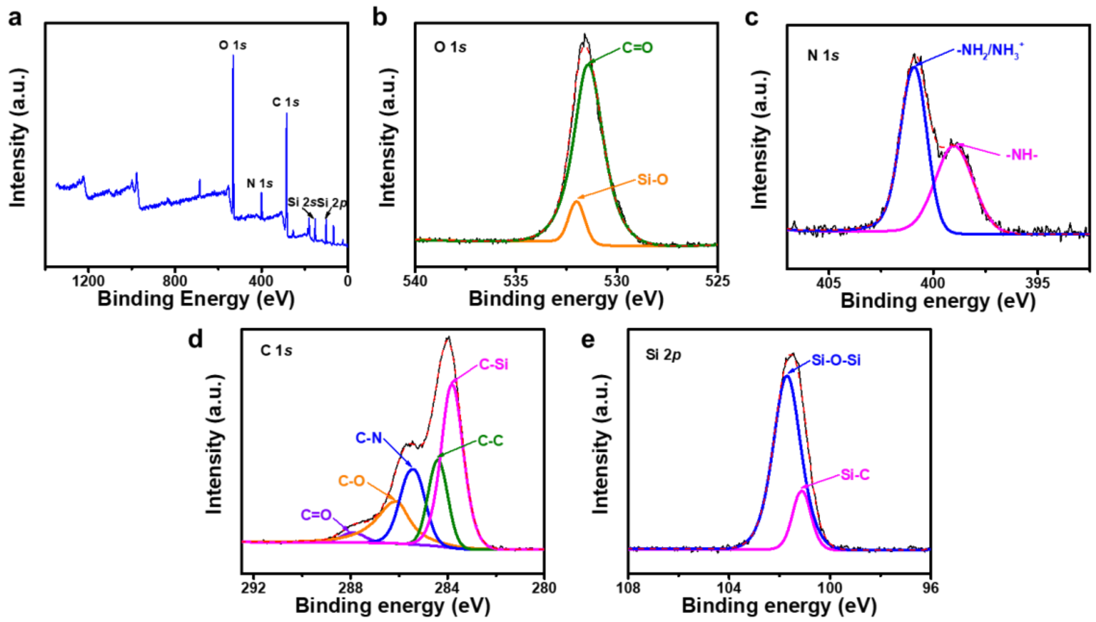

Supplementary Fig. 19. **a** XPS spectrum of the APTES-crosslinked rGO aerogel. **b** XPS O 1*s*, **c** N 1*s*, **d** C 1*s*, and **e** Si 2*p* spectra of the APTES-crosslinked rGO aerogel. Source data are provided as a Source Data file.

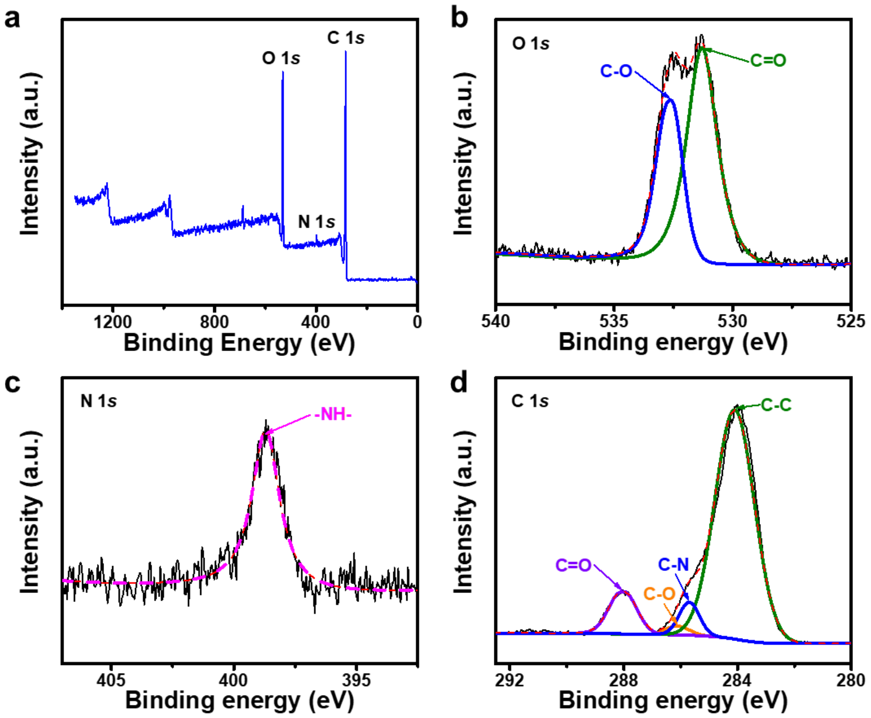

Supplementary Fig. 20. **a** XPS spectrum of the GO/PU aerogel without APTES. **b** XPS O 1*s*, **c** N 1*s*, and **d** C 1*s* spectra of the GO/PU aerogel without APTES. Source data are provided as a Source Data file.

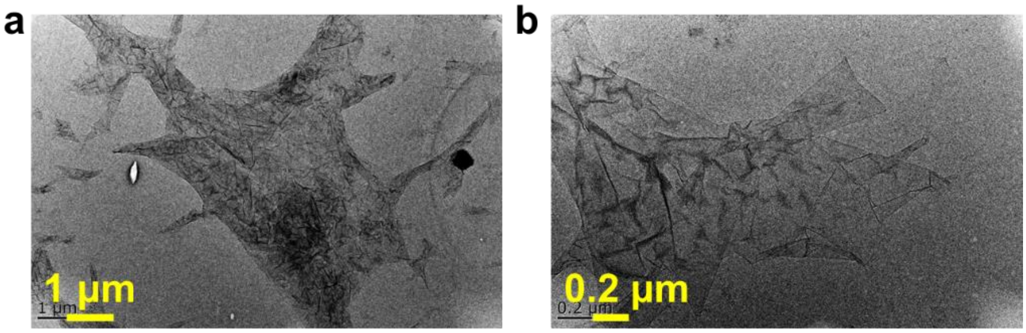

Supplementary Fig. 21. TEM images of GO sheets in the GO dispersion.

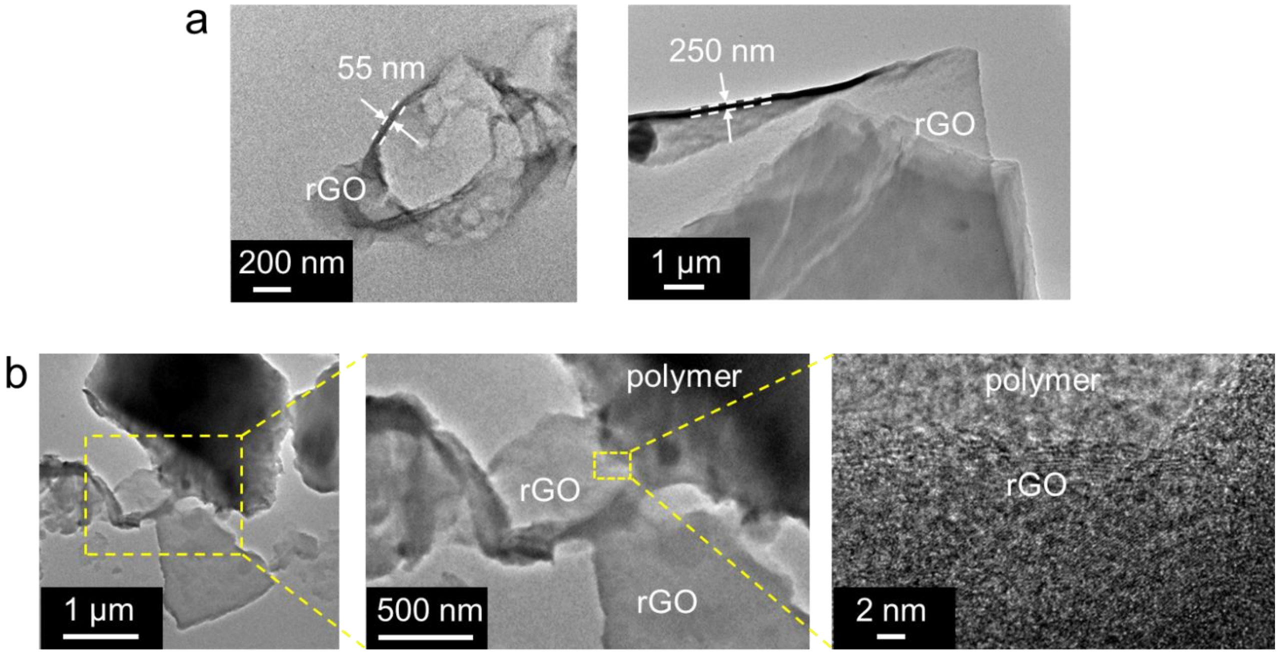

Supplementary Fig. 22. TEM images of rGO/PPy/PU-UHP. **a** rGO nanosheets. **b** Interface between rGO and polymer.

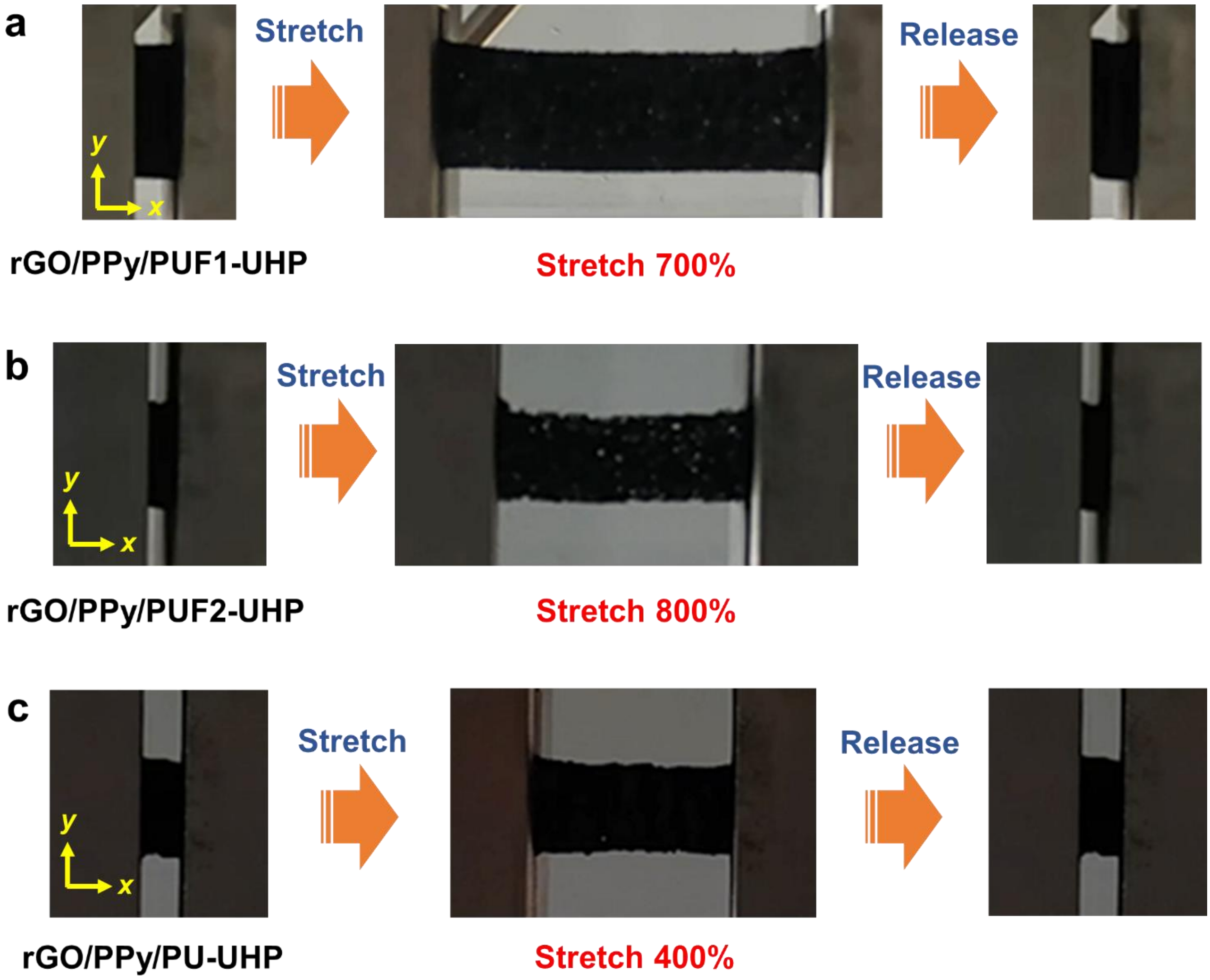

Supplementary Fig. 23. Photographs of the stretching-releasing tests on uniaxially hot-pressed rGO/polymer elastomers with different tensile strains in *x* direction. **a** rGO/PPy/PUF1-UHP. **b** rGO/PPy/PUF2-UHP. **c** rGO/PPy/PU-UHP. All these elastomers can be stretched with 400-800% tensile strains without fracture and recover their original shapes after being released, indicating the high stretchability and high elasticity of the uniaxially hot-pressed elastomers.

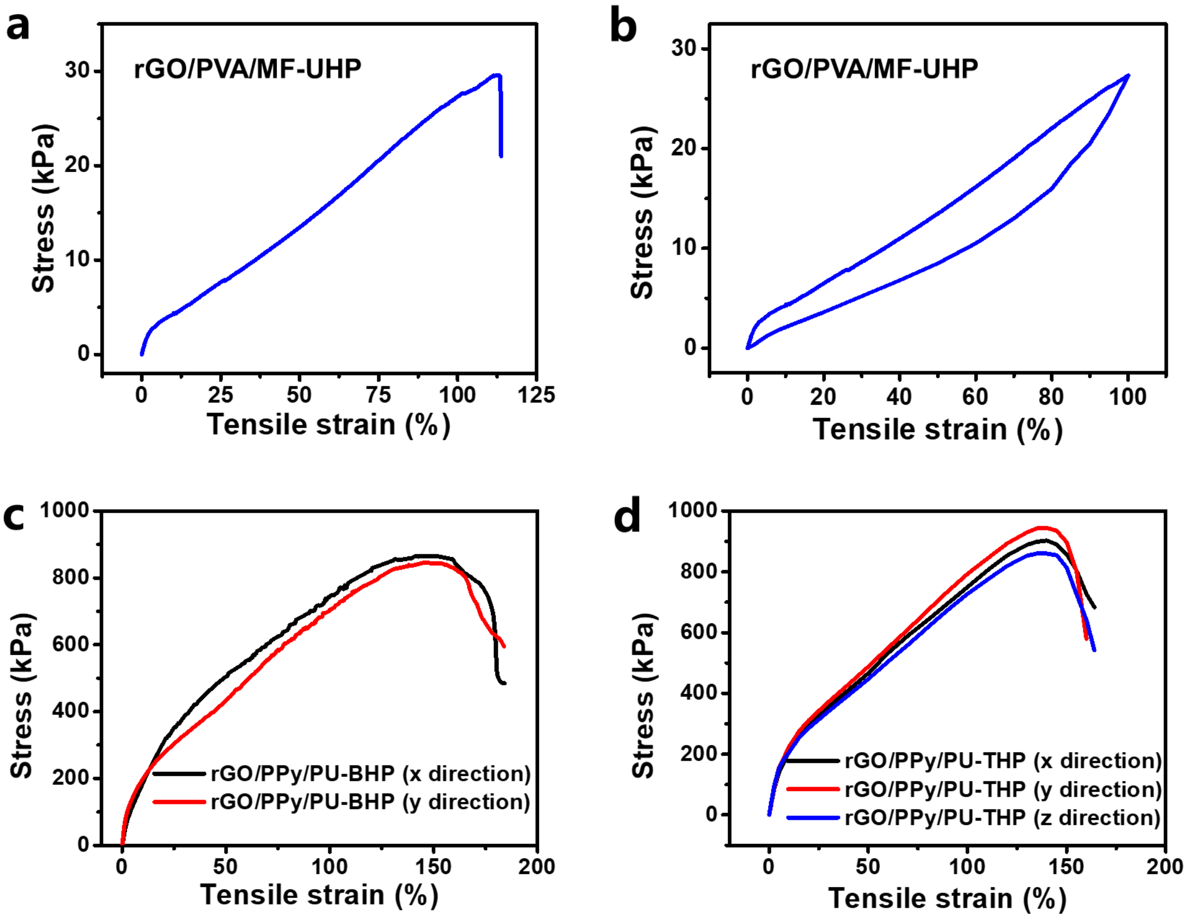

Supplementary Fig. 24. Stress-strain curves of the tensile tests on the uniaxially, biaxially, and triaxially hot-pressed elastomers. **a,b** Stress-strain curves of the tensile tests in *x* direction on rGO/PVA/MF-UHP. **c,d** Stress-strain curves of the tensile tests in different directions on **c** rGO/PPy/PU-BHP and **d** rGO/PPy/PU-THP. rGO/PVA/MF-UHP exhibits an elongation at break of 112% and can be reversibly stretched with 100% tensile strain. The elongations at break of rGO/PPy/PU-BHP and rGO/PPy/PU-THP are approximately 160% and 140%, respectively. Source data are provided as a Source Data file.

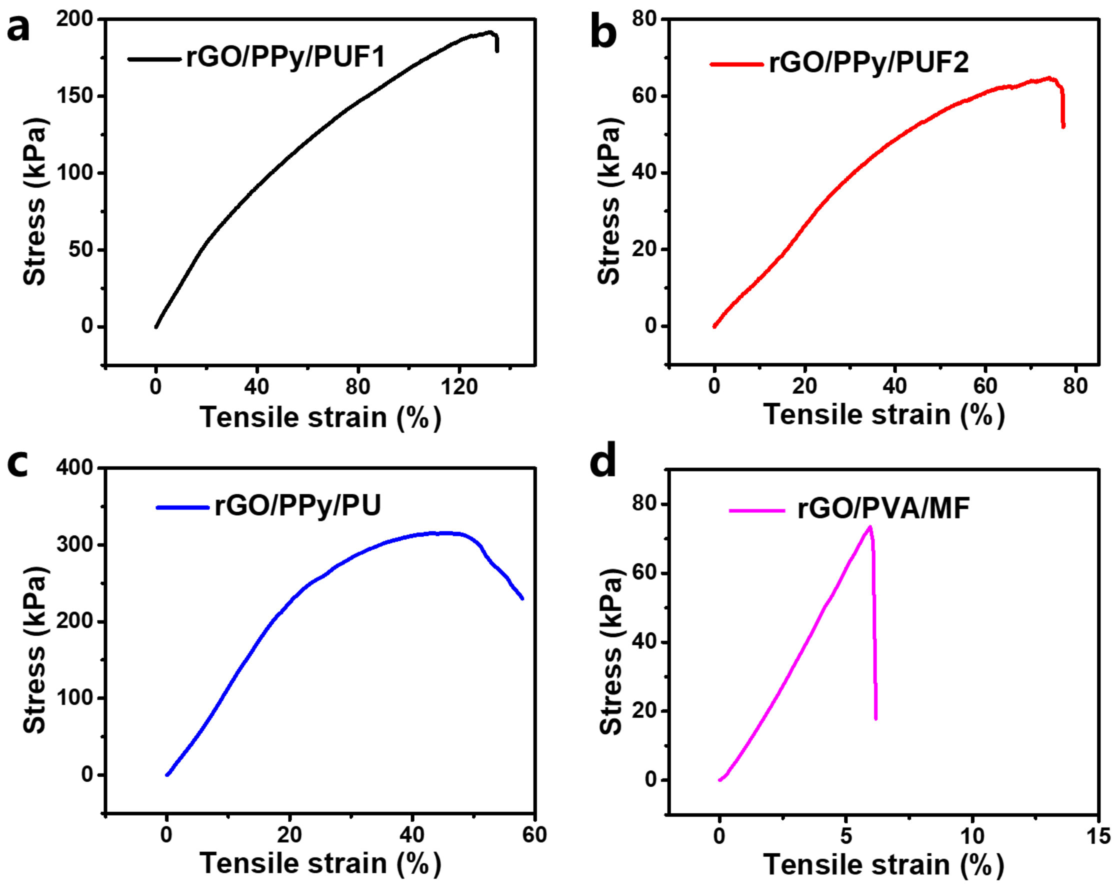

Supplementary Fig. 25. Stress-strain curves of the tensile tests on the pristine rGO/polymer aerogels without hot pressing. **a** rGO/PPy/PUF1. **b** rGO/PPy/PUF2. **c** rGO/PPy/PU. **d** rGO/PVA/MF. The elongations at break of rGO/PPy/PUF1, rGO/PPy/PUF2, rGO/PPy/PU, and rGO/PVA/MF are 130%, 76%, 51%, and 6%, respectively. Source data are provided as a Source Data file.

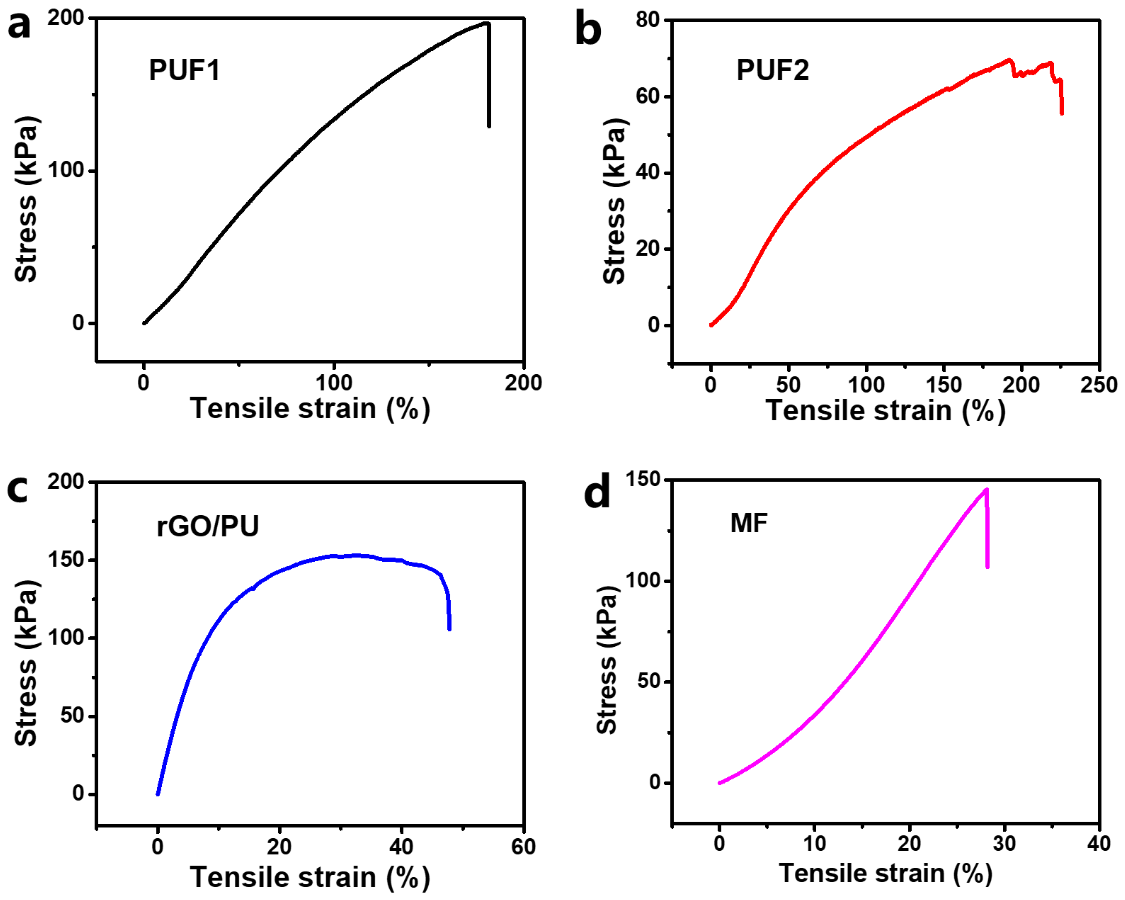

Supplementary Fig. 26. Stress-strain curves of the tensile tests on the foams and rGO/PU aerogel. **a** PUF1. **b** PUF2. **c** rGO/PU aerogel. **d** MF. The elongations at break of PUF1, PUF2, MF, and the rGO/PU aerogel are 180%, 193%, 28%, and 47%, respectively. Source data are provided as a Source Data file.

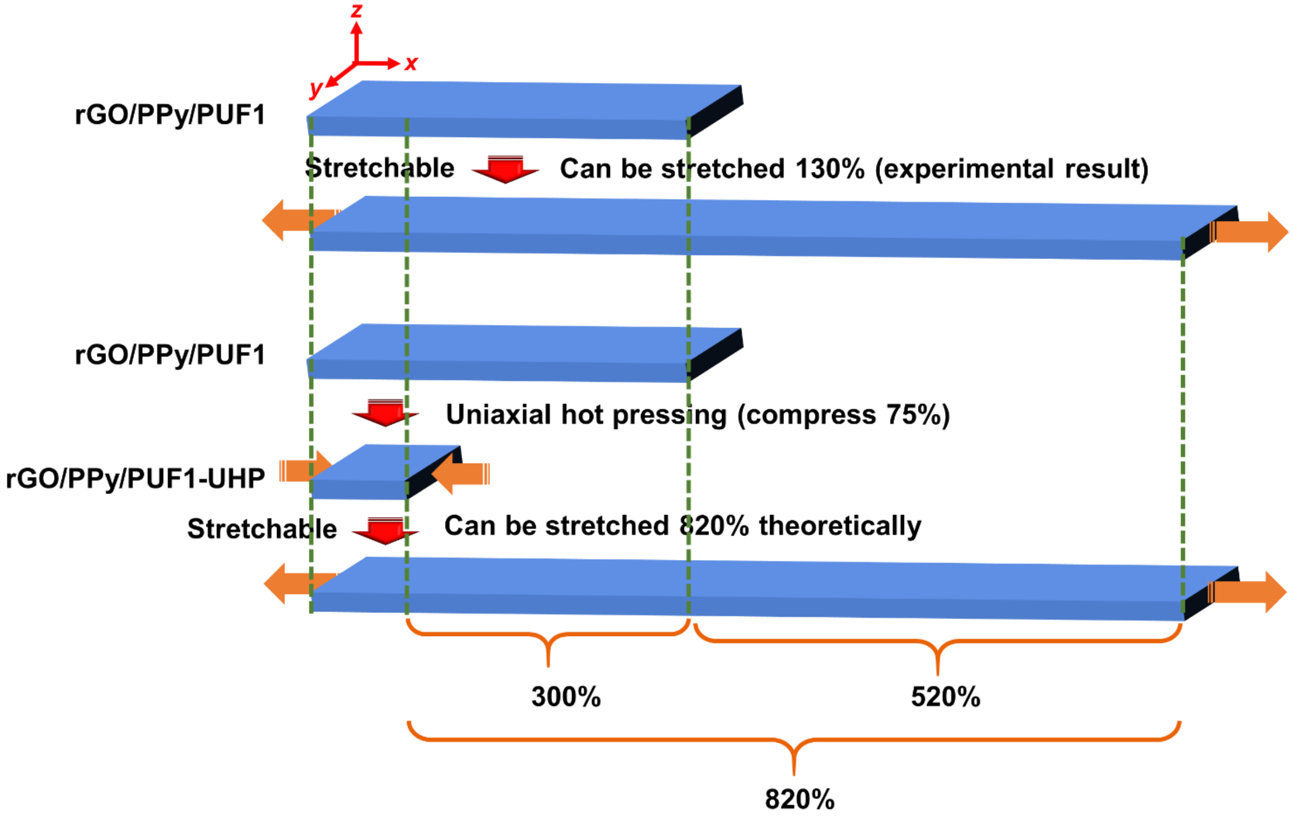

Supplementary Fig. 27. Illustration showing the calculation of the theoretical value of the elongation at break for the uniaxially hot-pressed elastomer rGO/PPy/PUF1-UHP.

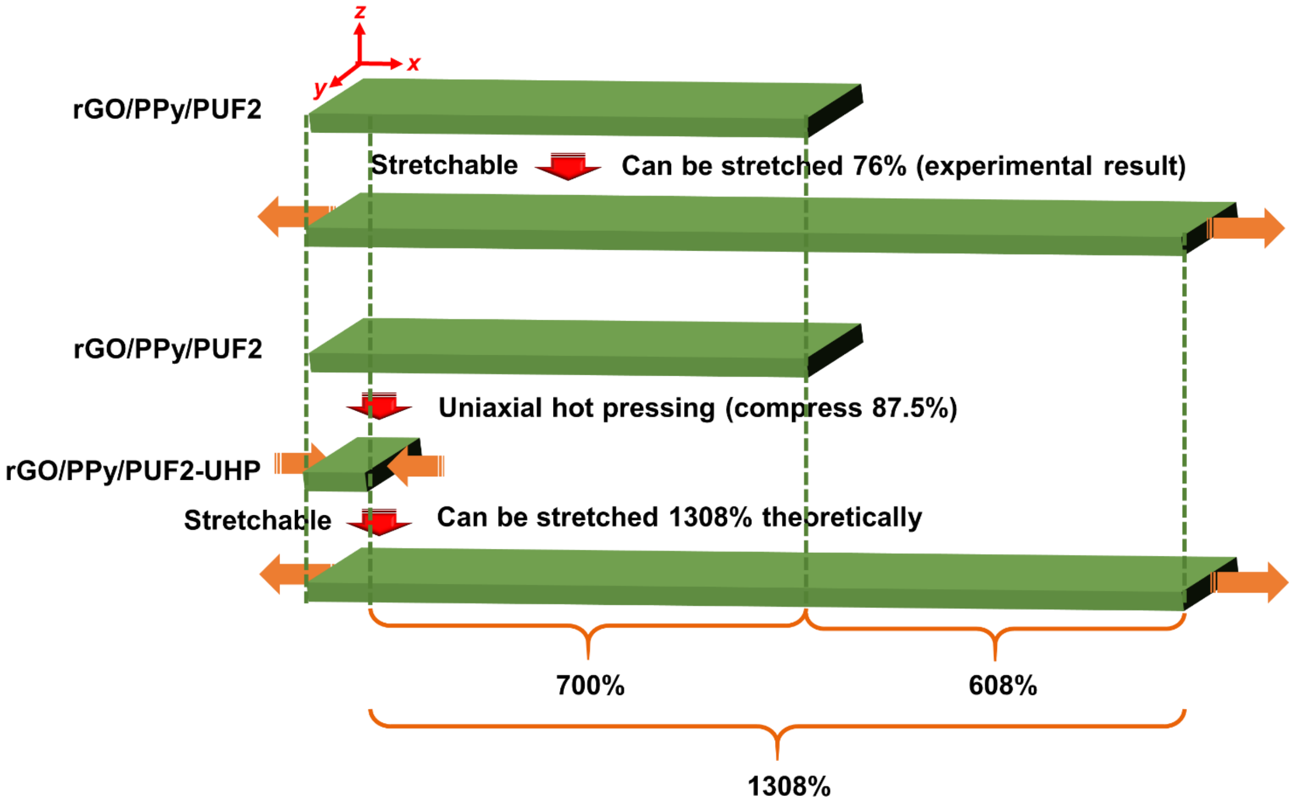

Supplementary Fig. 28. Illustration showing the calculation of the theoretical value of the elongation at break for the uniaxially hot-pressed elastomer rGO/PPy/PUF2-UHP.

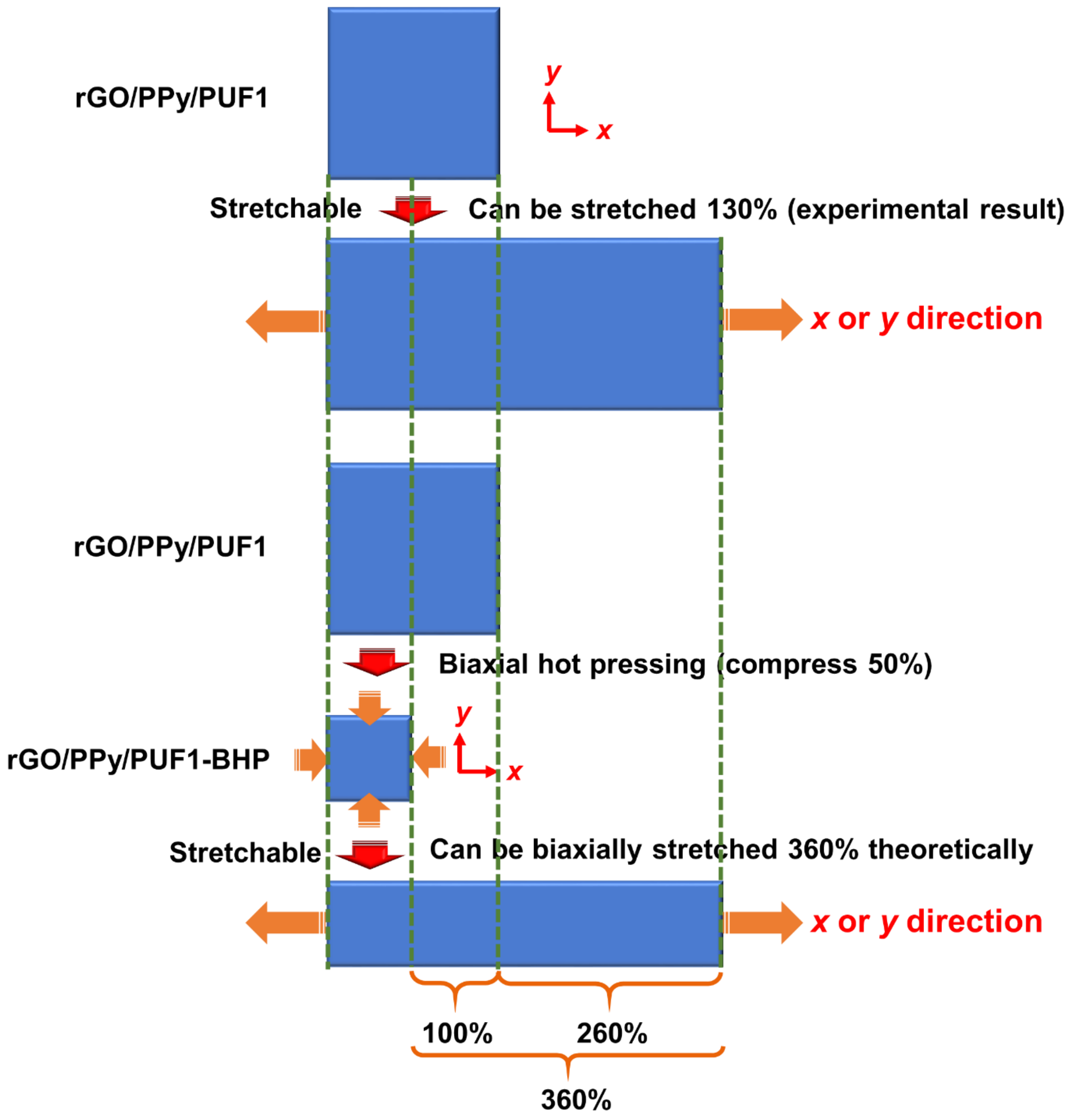

Supplementary Fig. 29. Illustration showing the calculation of the theoretical value of the elongation at break for the biaxially hot-pressed elastomer rGO/PPy/PUF1-BHP.

a

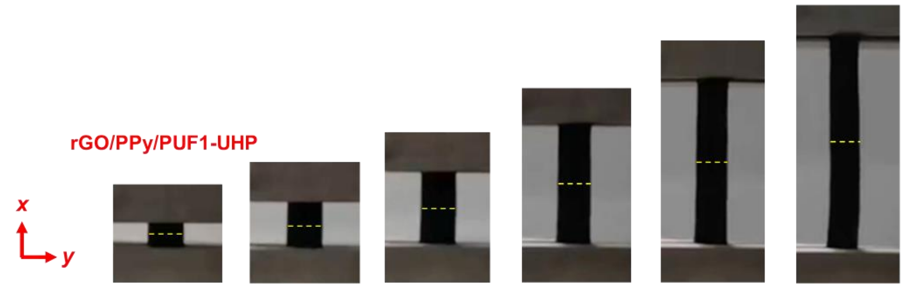

ε_y/%	0	0	-1.5	-6.0	-9.8	-13.1
ε_x/%	0	100	200	400	600	800
ν	0	0	0.007	0.015	0.016	0.016

b

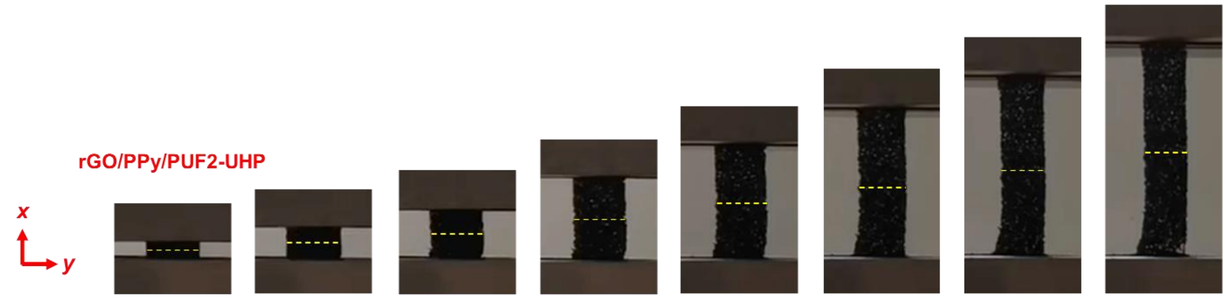

ε_y/%	0	0	0	-1.5	-3.0	-10.0	-15.0	-17.5
ε_x/%	0	100	200	400	600	800	1000	1200
ν	0	0	0	0.004	0.005	0.012	0.015	0.014

c

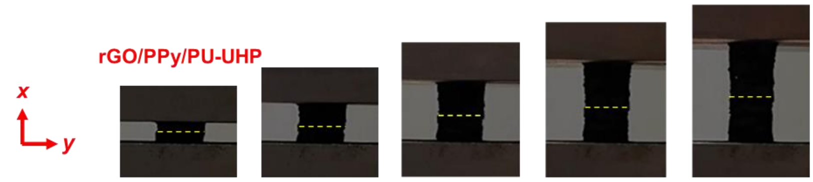

ε_y/%	0	0	-2.2	-4.3	-5.5
ε_x/%	0	100	200	300	400
ν	0	0	0.011	0.014	0.014

Supplementary Fig. 30. Photographs and Poisson's ratios (ν) of the uniaxially hot-pressed rGO/polymer elastomers upon stretching with different tensile strains in *x* direction. **a** rGO/PPy/PUF1-UHP. **b** rGO/PPy/PUF2-UHP. **c** rGO/PPy/PU-UHP. The uniaxially hot-pressed elastomers showed zero or low Poisson's ratios upon stretching over a wide range of tensile strains.

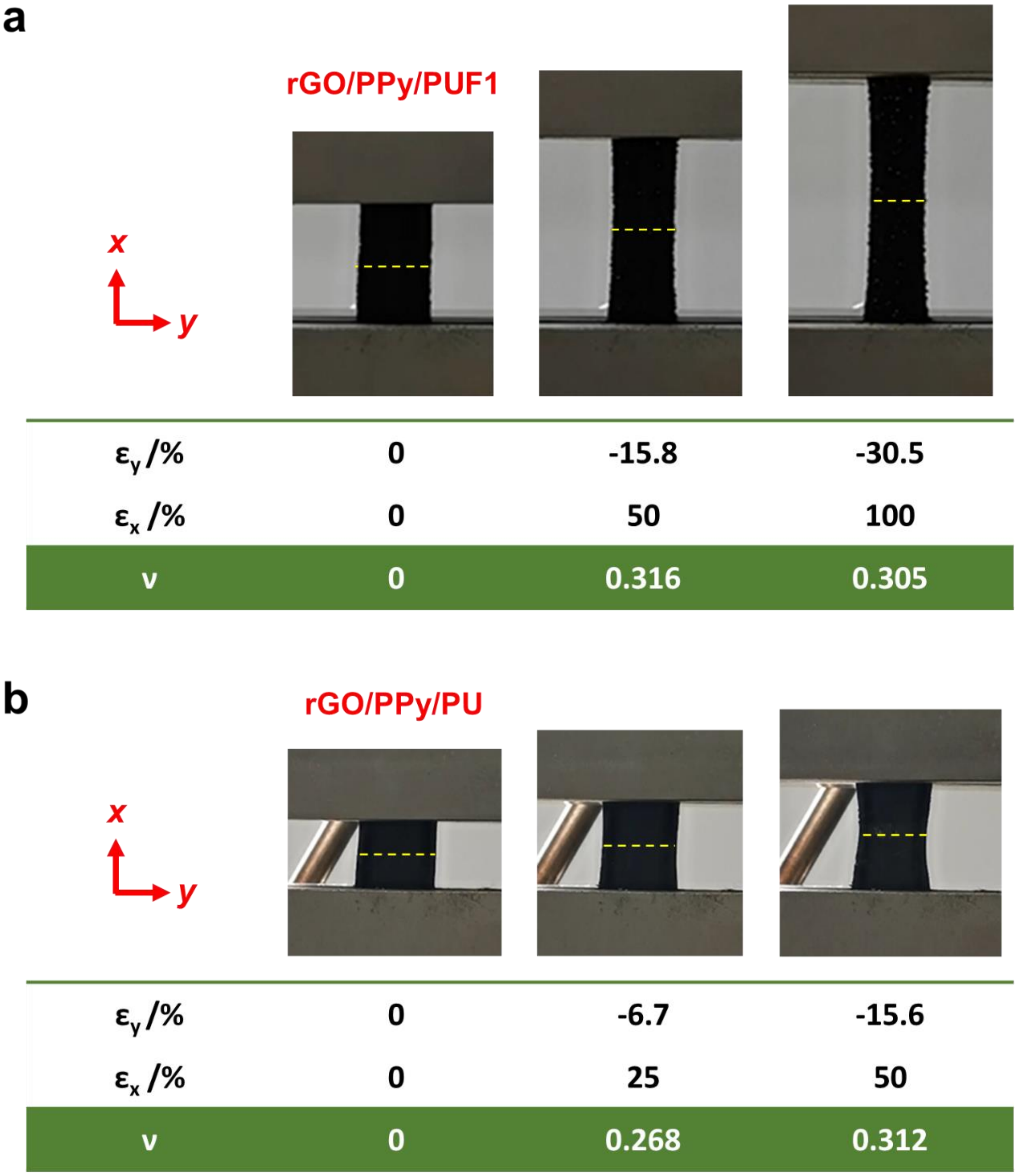

Supplementary Fig. 31. Photographs and Poisson's ratios (ν) of the rGO/polymer aerogels without hot pressing upon stretching. **a** rGO/PPy/PUF1. **b** rGO/PPy/PU. It is shown that the Poisson's ratios of the rGO/polymer aerogels without hot pressing are much higher than those of the uniaxially hot-pressed rGO/polymer elastomers upon stretching in *x* direction.

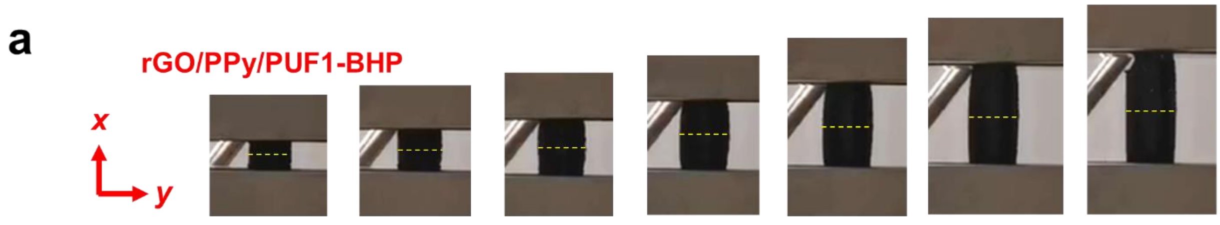

ε_y /%	0	8.7	15.9	23.2	23.2	21.7	21.7
ε_x /%	0	50	100	150	200	250	300
ν	0	-0.174	-0.159	-0.154	-0.116	-0.087	-0.072

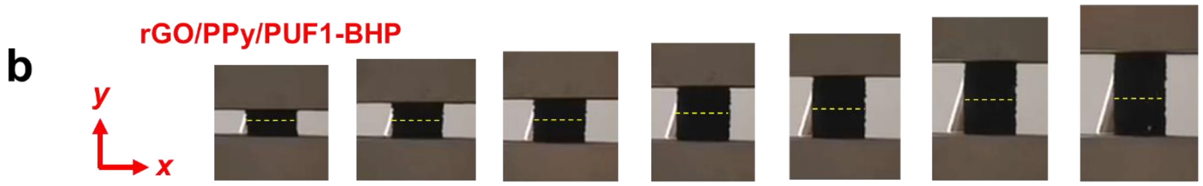

ε_x /%	0	6.0	9.6	13.3	10.8	10.8	9.6
ε_y /%	0	50	100	150	200	250	300
ν	0	-0.120	-0.096	-0.089	-0.054	-0.043	-0.032

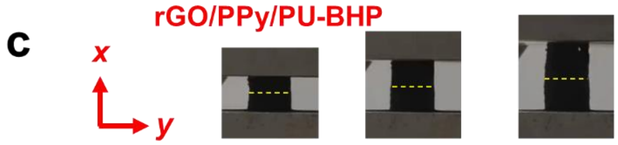

ε_y /%	0	5.5	9.1
ε_x /%	0	50	100
ν	0	-0.110	-0.091

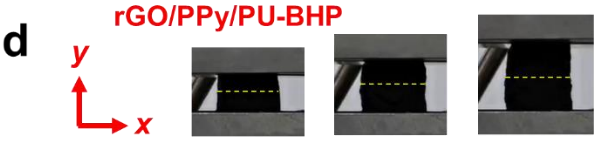

ε_x /%	0	5.3	5.9
ε_y /%	0	50	100
ν	0	-0.106	-0.059

Supplementary Fig. 32. Photographs and Poisson's ratios (ν) of the biaxially hot-pressed rGO/polymer elastomers upon stretching in *x* and *y* directions with different tensile strains. **a** rGO/PPy/PUF1-BHP stretched in *x* direction. **b** rGO/PPy/PUF1-BHP stretched in *y* direction. **c** rGO/PPy/PU-BHP stretched in *x* direction. **d** rGO/PPy/PU-BHP stretched in *y* direction. The biaxially hot-pressed rGO/polymer elastomers showed negative Poisson's ratios upon stretching in both *x* and *y* directions over a wide range of tensile strains.

a

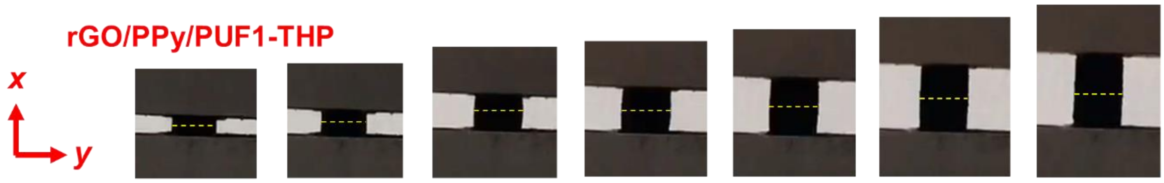

ε_y /%	0	5.3	15.8	15.8	14.7	12.6	12.6
ε_x /%	0	50	100	150	200	250	300
v	0	-0.106	-0.158	-0.105	-0.074	-0.050	-0.042

b

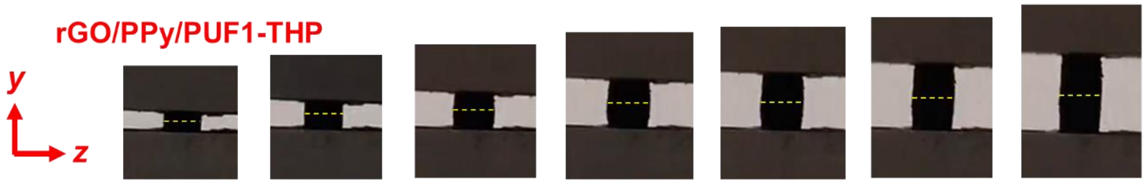

ε_z /%	0	6.3	15.0	20.0	20.0	17.5	17.5
ε_y /%	0	50	100	150	200	250	300
v	0	-0.126	-0.150	-0.133	-0.100	-0.070	-0.058

c

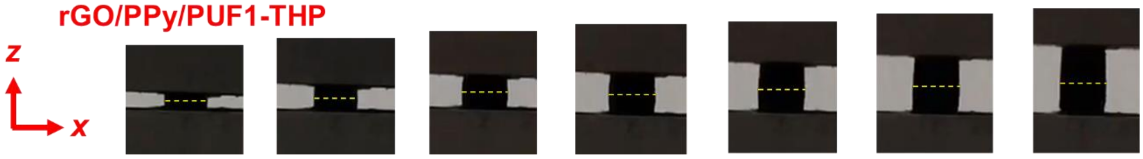

ε_x /%	0	4.3	11.8	14.0	14.0	14.0	11.8
ε_z /%	0	50	100	150	200	250	300
v	0	-0.086	-0.118	-0.093	-0.070	-0.056	-0.039

Supplementary Fig. 33. Photographs and Poisson's ratios (ν) of the triaxially hot-pressed rGO/polymer elastomer rGO/PPy/PUF1-THP upon stretching in *x*, *y*, and *z* directions with different tensile strains. **a** Stretch in *x* direction. **b** Stretch in *y* direction. **c** Stretch in *z* direction. The triaxially hot-pressed rGO/polymer elastomer showed negative Poisson's ratios upon stretching in *x, y*, and *z* directions over a wide range of tensile strains.

a

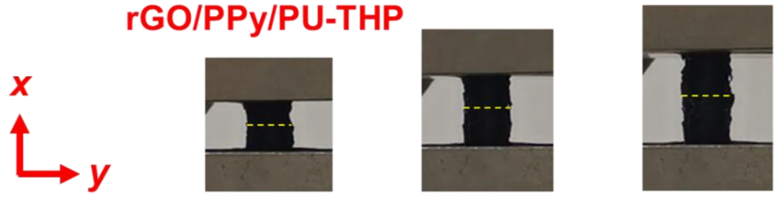

ε_y /%	0	5.0	12.0
ε_x /%	0	50	100
ν	0	-0.100	-0.120

b

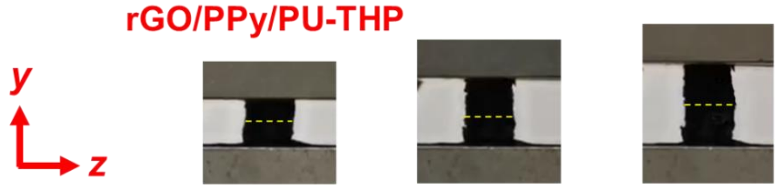

ε_z /%	0	4.8	9.5
ε_y /%	0	50	100
ν	0	-0.096	-0.095

c

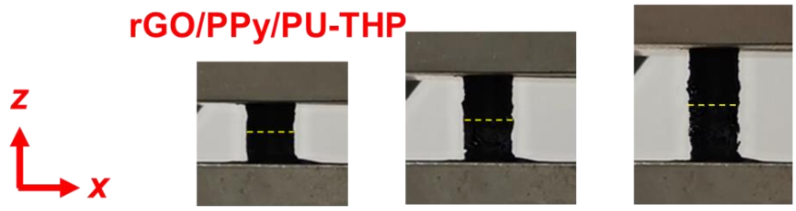

ε_x /%	0	4.7	6.7
ε_z /%	0	50	100
ν	0	-0.094	-0.067

Supplementary Fig. 34. Photographs and Poisson's ratios (ν) of the triaxially hot-pressed rGO/polymer elastomer rGO/PPy/PU-THP upon stretching in *x*, *y*, and *z* directions with different tensile strains. **a** Stretch in *x* direction. **b** Stretch in *y* direction. **c** Stretch in *z* direction. The triaxially hot-pressed rGO/polymer elastomer rGO/PPy/PU-THP showed negative Poisson's ratios upon stretching in *x, y*, and *z* directions.

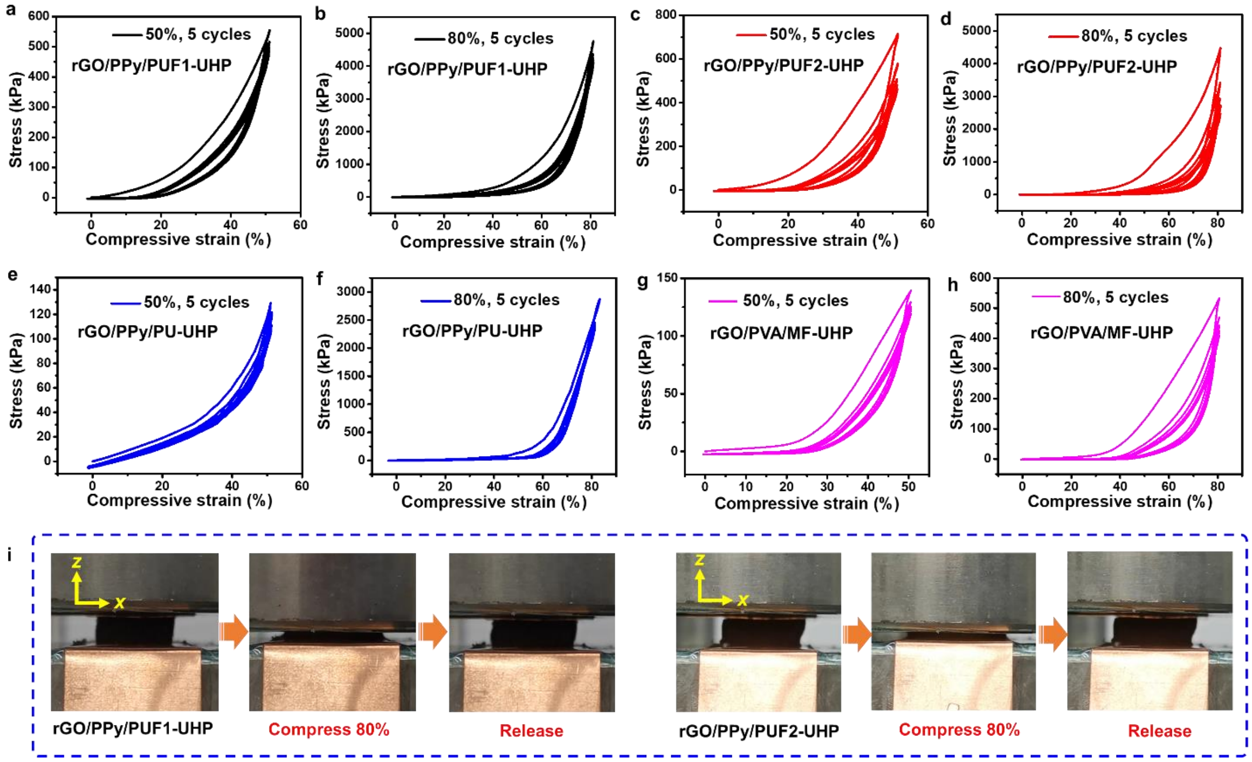

Supplementary Fig. 35. Stress-strain curves and photographs of the compression-decompression tests in *z* direction on the uniaxially hot-pressed rGO/polymer elastomers with strains of 50% and 80%. **a,b** rGO/PPy/PUF1-UHP. **c,d** rGO/PPy/PUF2-UHP. **e,f** rGO/PPy/PU-UHP. **g,h** rGO/PVA/MF-UHP. **i** Photographs of the compression-decompression tests on rGO/PPy/PUF1-UHP and rGO/PPy/PUF2-UHP. The compressive stresses of rGO/PPy/PUF1-UHP, rGO/PPy/PUF2-UHP, rGO/PPy/PU-UHP, and rGO/PVA/MF-UHP at 80% strain in *z* direction are 4.7 MPa, 4.4 MPa, 2.8 MPa, and 530 kPa, respectively. Source data are provided as a Source Data file.

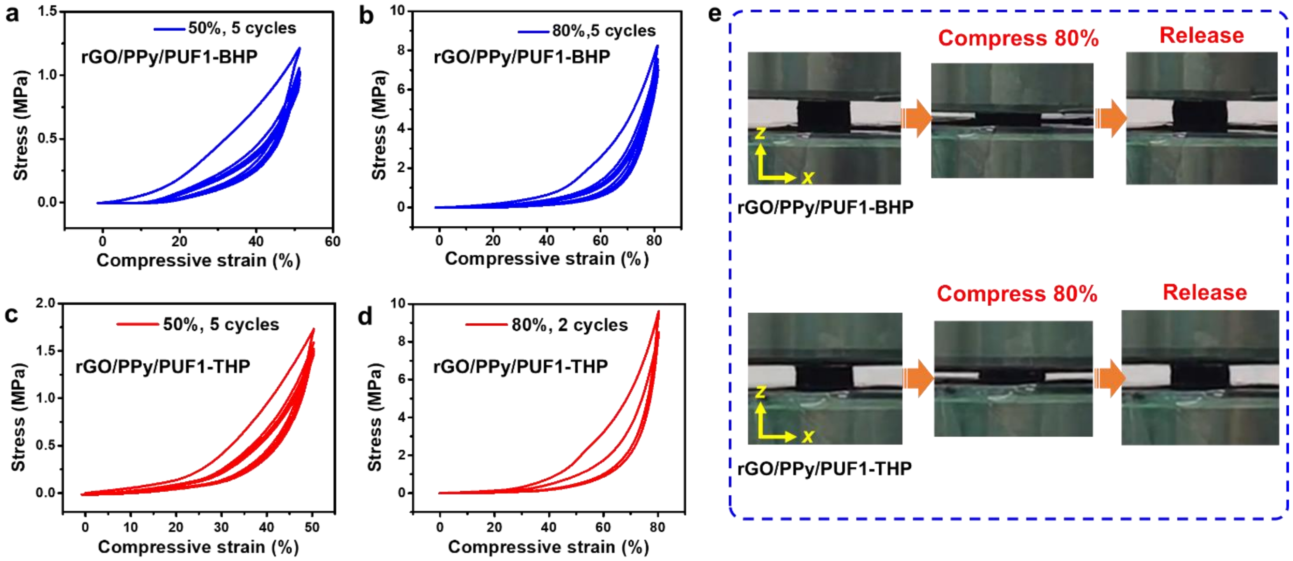

Supplementary Fig. 36. Compression-decompression tests in *z* direction on biaxially and triaxially hot-pressed rGO/polymer elastomers with strains of 50% and 80%. **a,b** rGO/PPy/PUF1-BHP. **c,d** rGO/PPy/PUF1-THP. **e** Photographs of the compression-decompression tests on rGO/PPy/PUF1-BHP and rGO/PPy/PUF1-THP. The compressive stresses of rGO/PPy/PUF1-BHP and rGO/PPy/PUF1-THP at 80% strain in *z* direction are 8.2 MPa and 9.5 MPa, respectively. Source data are provided as a Source Data file.

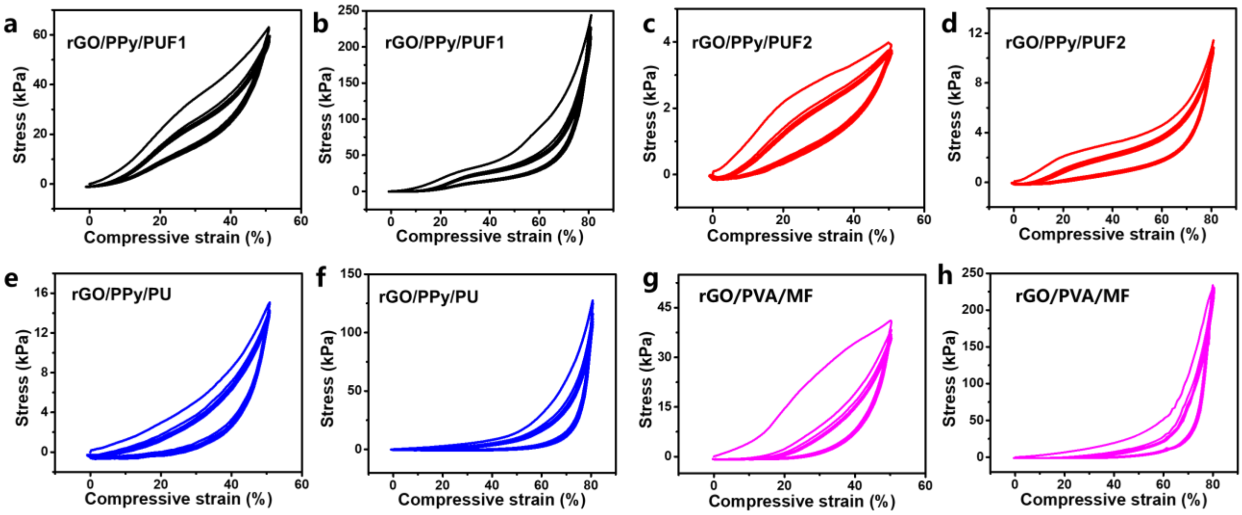

Supplementary Fig. 37. Stress-strain curves of the compression-decompression tests (5 cycles) in *z* direction on the pristine rGO/polymer aerogels without hot pressing with strains of 50% and 80%. **a,b** rGO/PPy/PUF1. **c,d** rGO/PPy/PUF2. **e,f** rGO/PPy/PU. **g,h** rGO/PVA/MF. The compressive stresses of

rGO/PPy/PUF1, rGO/PPy/PUF2, rGO/PPy/PU, and rGO/PVA/MF at 80% strain are 242 kPa, 11.5 kPa, 126 kPa, and 225 kPa, respectively. Source data are provided as a Source Data file.

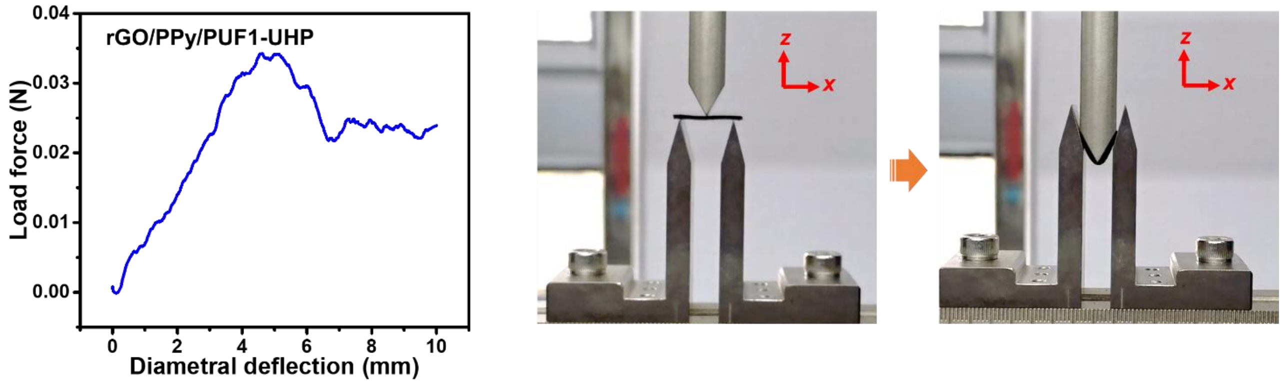

Supplementary Fig. 38. Three-point bending test on the uniaxially hot-pressed elastomer rGO/PPy/PUF1-UHP. Source data are provided as a Source Data file.

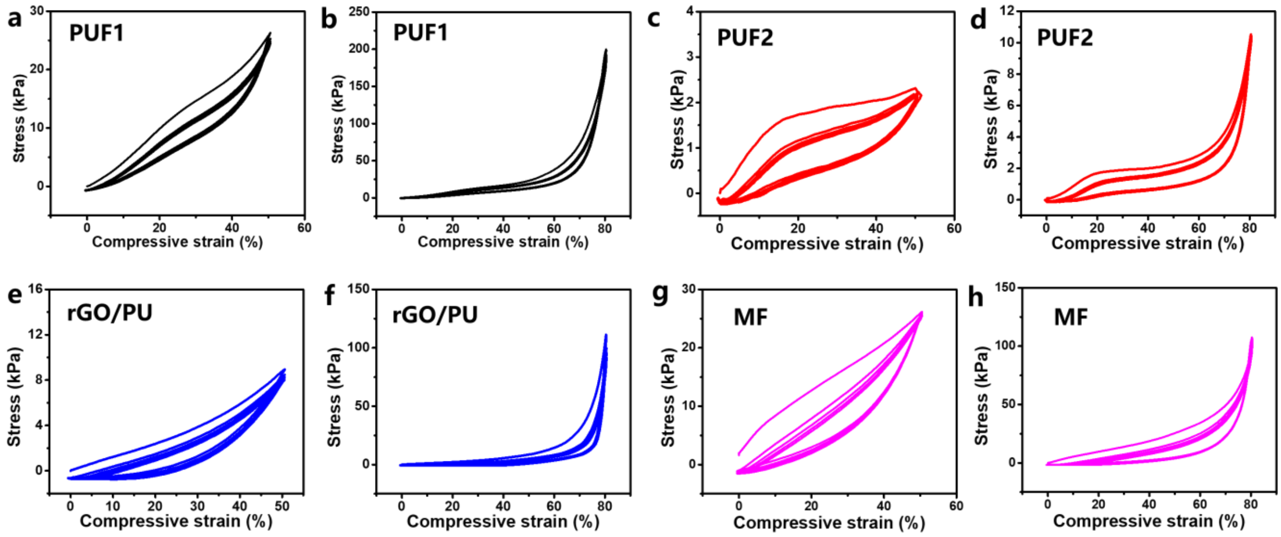

Supplementary Fig. 39. Stress-strain curves of the compression-decompression tests (5 cycles) in *z* direction on the foams and the rGO/PU aerogel with strains of 50% and 80%. **a,b** PUF1. **c,d** PUF2. **e,f** rGO/PU aerogel. **g,h** MF. Source data are provided as a Source Data file.

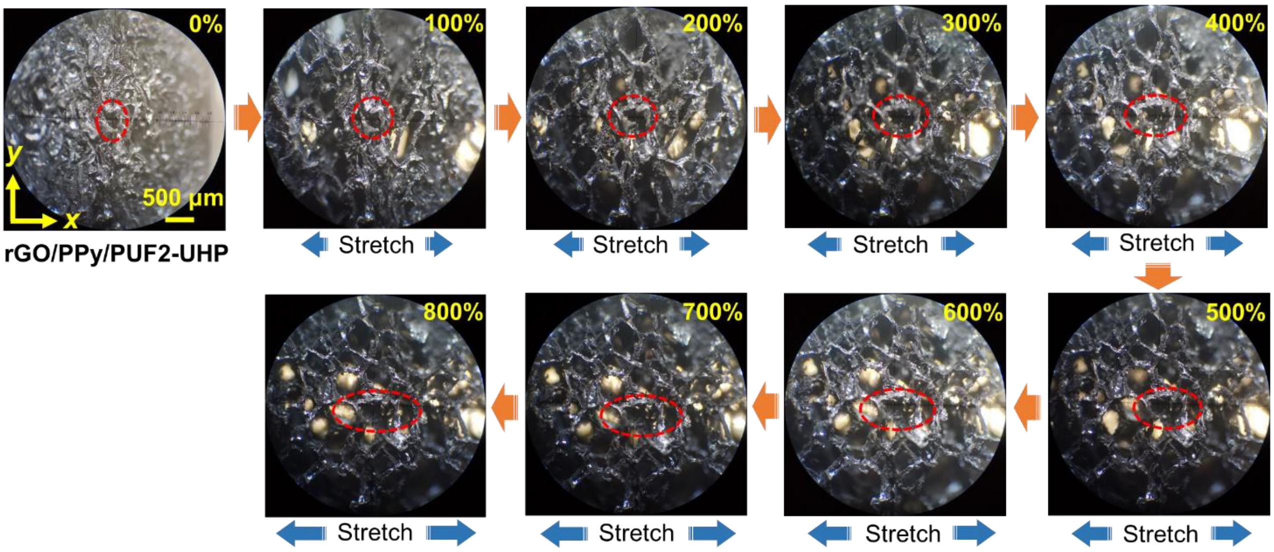

Supplementary Fig. 40. In-situ optical microscope images of rGO/PPy/PUF2-UHP upon stretching in *x* direction in the range of 0-800% tensile strain.

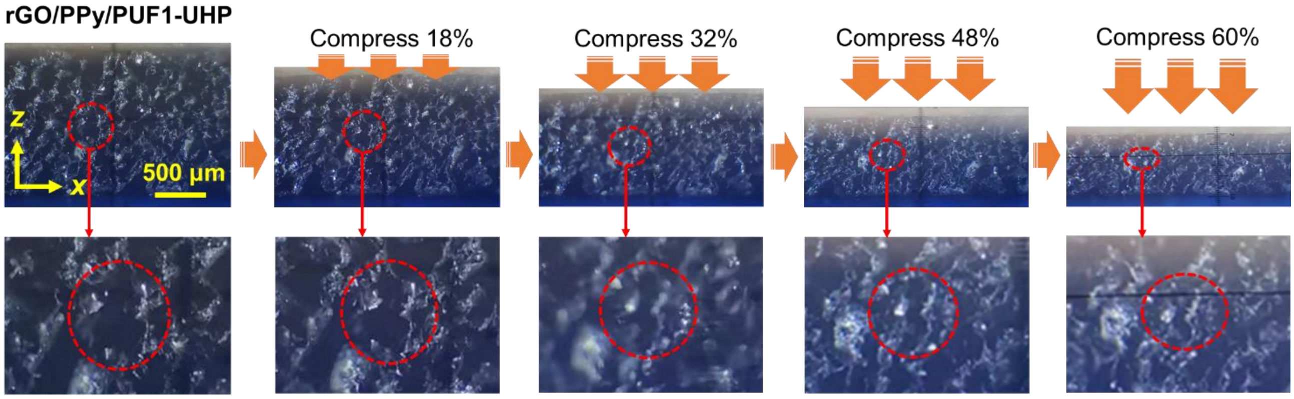

Supplementary Fig. 41. In-situ optical microscope images of rGO/PPy/PUF1-UHP upon compression in *z* direction in the range of 0-60% compressive strain.

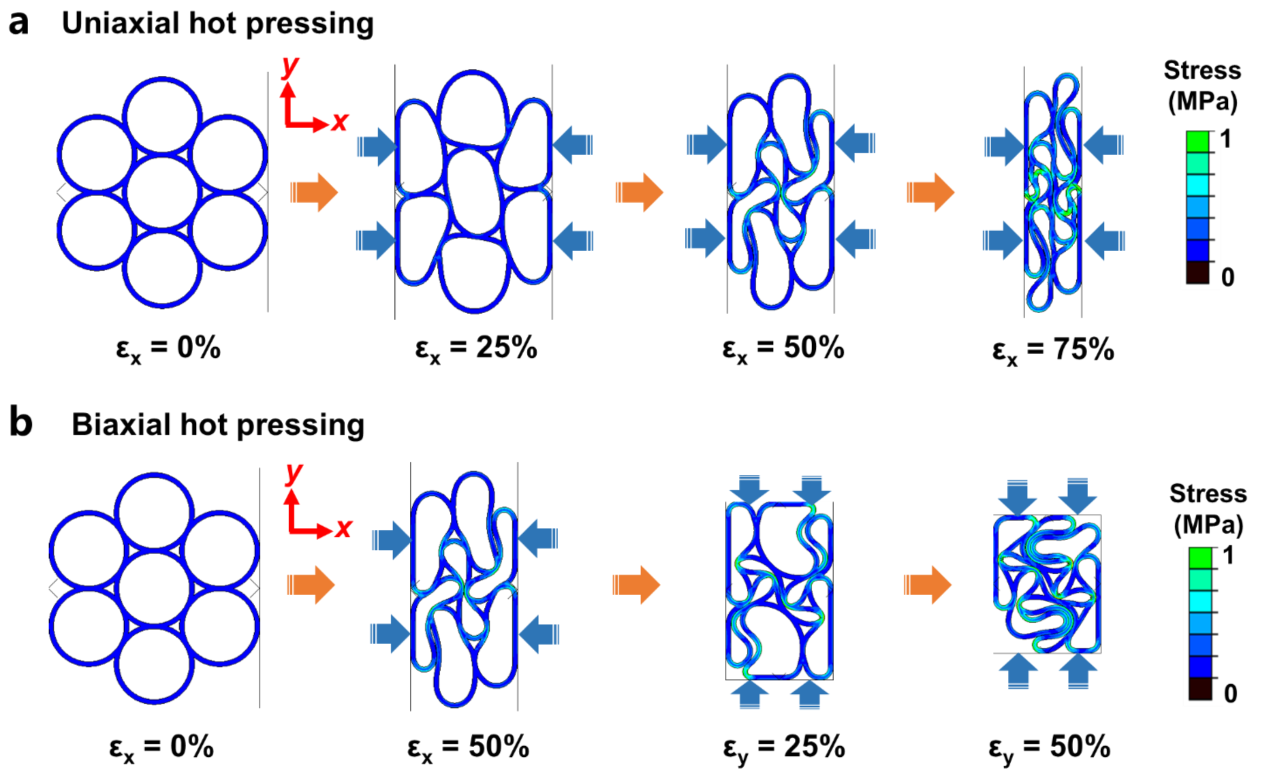

Supplementary Fig. 42. FEA simulations of the structure variations and stress distributions of the rGO/polymer composite elastomers during **a** uniaxial and **b** biaxial hot pressing.

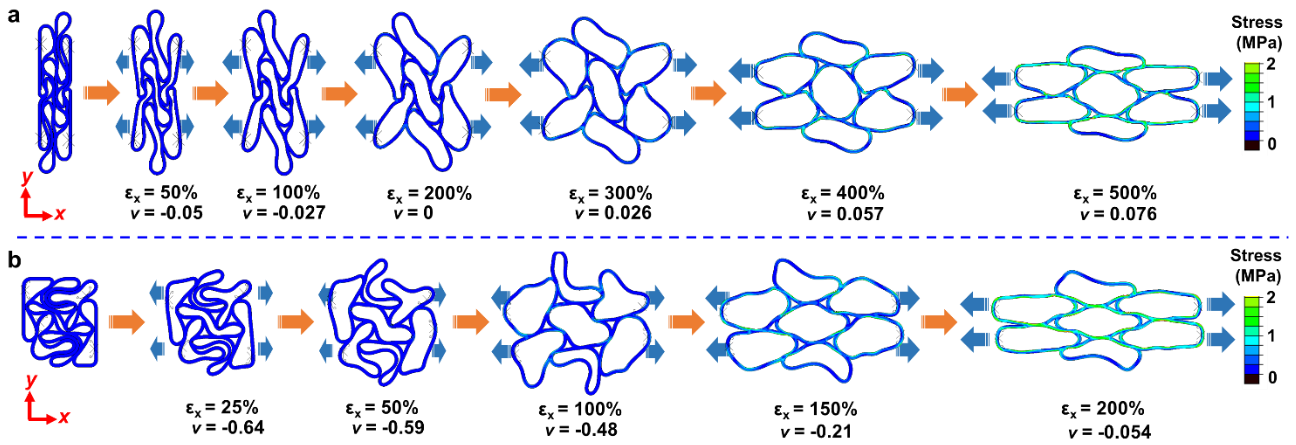

Supplementary Fig. 43. FEA simulations of the structure variations and stress distributions of the **a** uniaxially and **b** biaxially hot-pressed rGO/polymer composite elastomers during stretching.

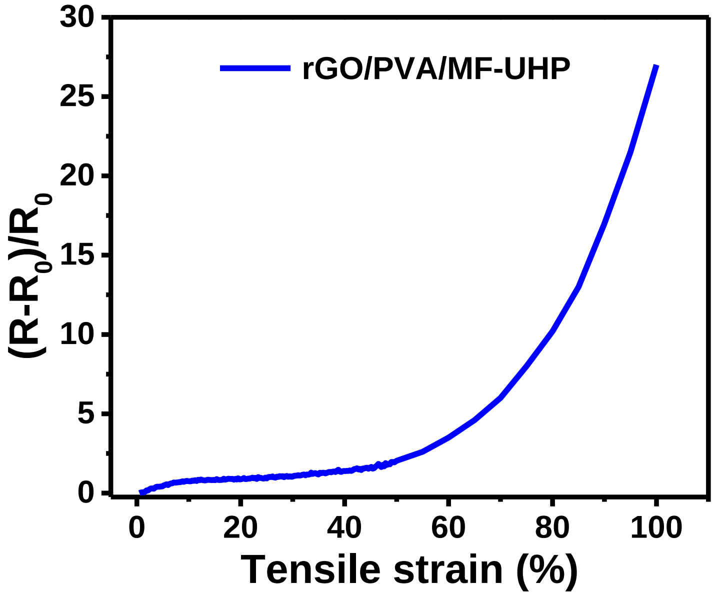

Supplementary Fig. 44. Relative resistance changes versus tensile strain in *x* direction for the strain sensor based on rGO/PVA/MF-UHP. Source data are provided as a Source Data file.

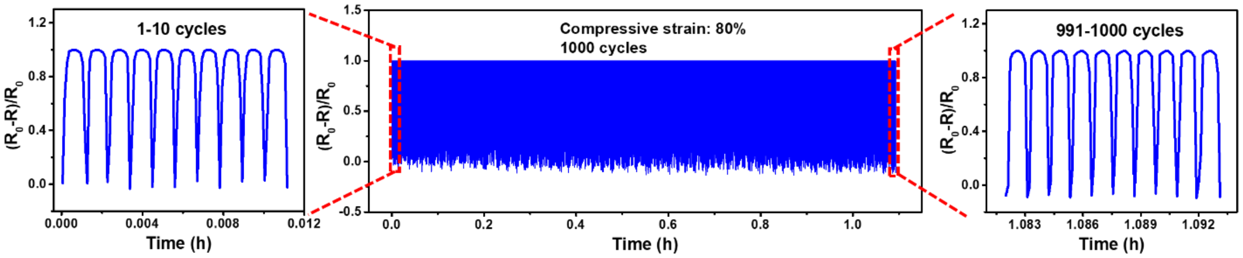

Supplementary Fig. 45. Compression durability test of the rGO/PPy/PUF1-UHP-based pressure sensor with 80% compressive strain in *z* direction for 1000 cycles. Source data are provided as a Source Data file.

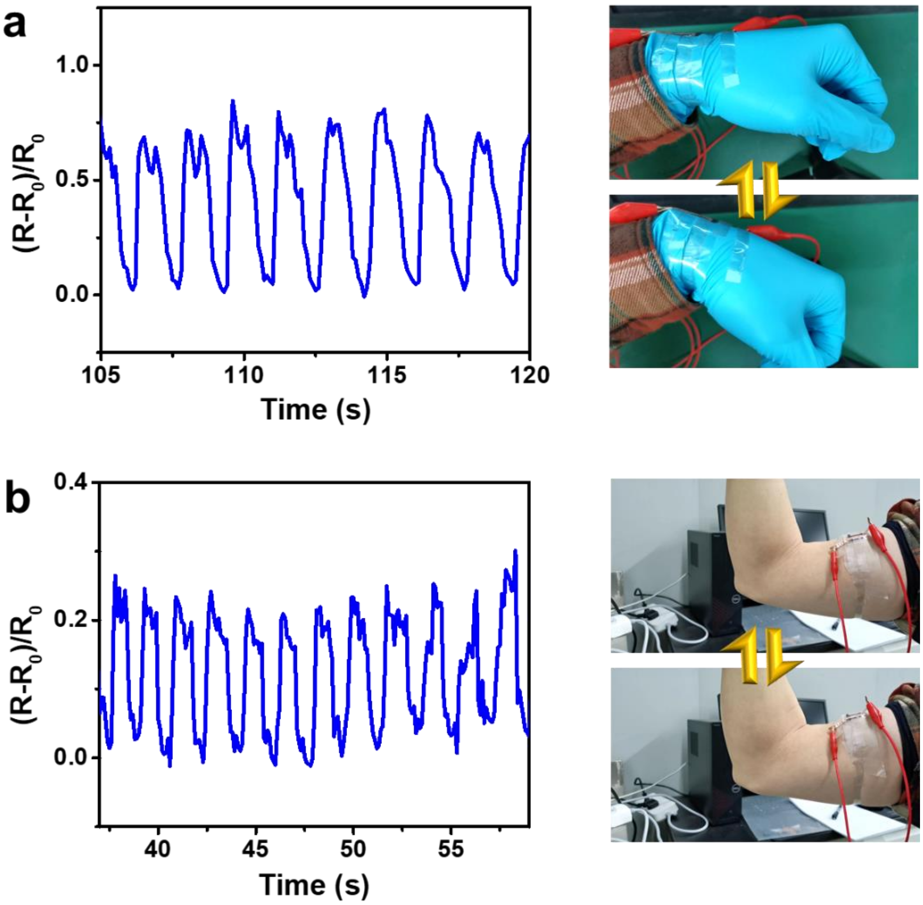

Supplementary Fig. 46. Monitoring **a** wrist bending and **b** muscular movement by the rGO/PPy/PUF1-UHP-based strain/pressure sensors. Source data are provided as a Source Data file.

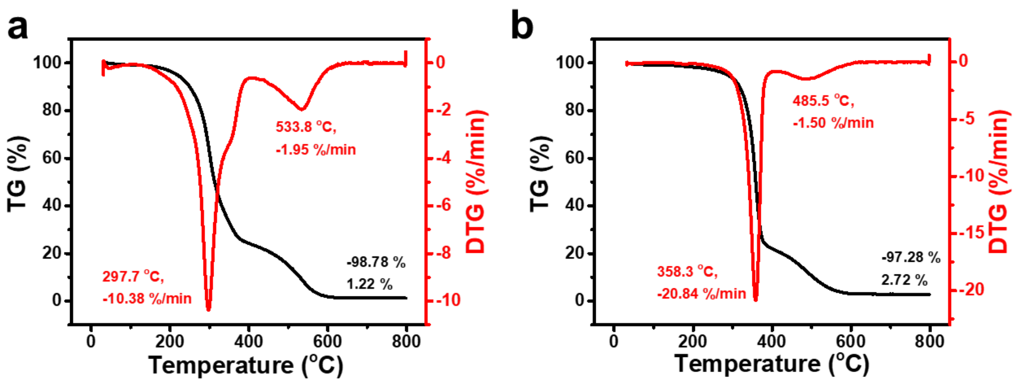

Supplementary Fig. 47. TG and DTG curves of a) rGO/PPy/PUF1-UHP and b) rGO/PPy/PU-UHP. Source data are provided as a Source Data file.

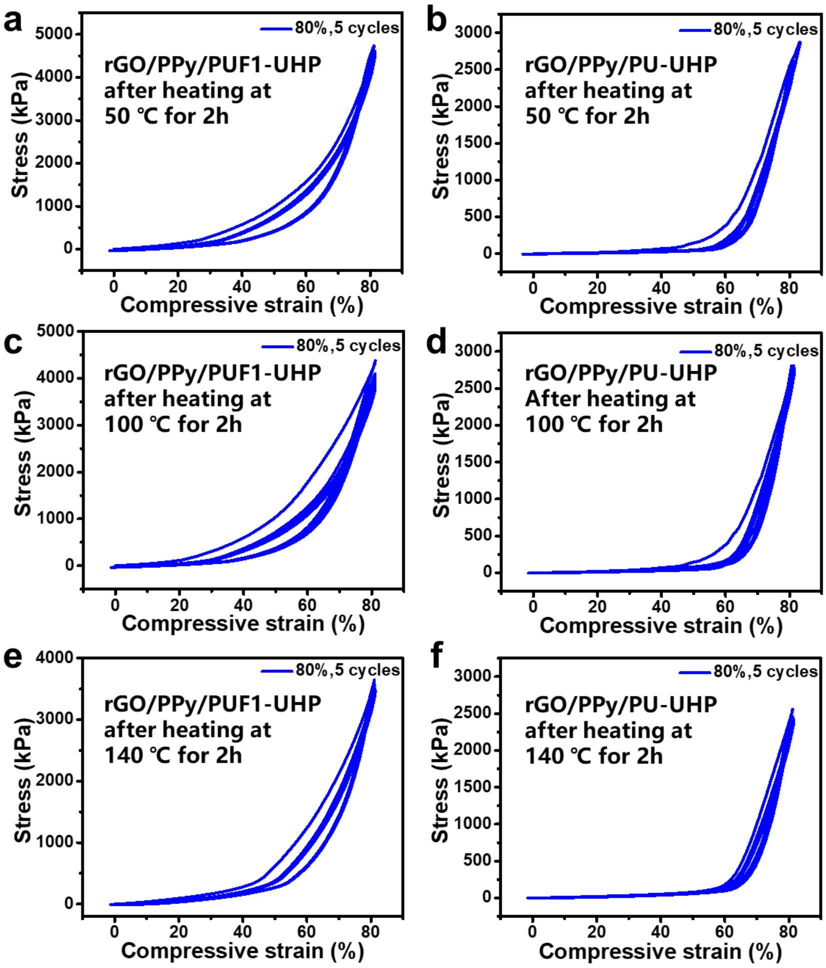

Supplementary Fig. 48. Stress-strain curves of typical hot-pressed elastomers with 80% compressive strain in *z* direction. a, c, e) Stress-strain curves of rGO/PPy/PUF1-UHP after heat treatment at a) 50 °C, c) 100 °C, and e) 140 °C for 2 h, respectively. b, d, f) Stress-strain curves of rGO/PPy/PU-UHP after heat treatment at b) 50 °C, d) 100 °C, and f) 140 °C for 2 h, respectively. Source data are provided as a Source Data file.

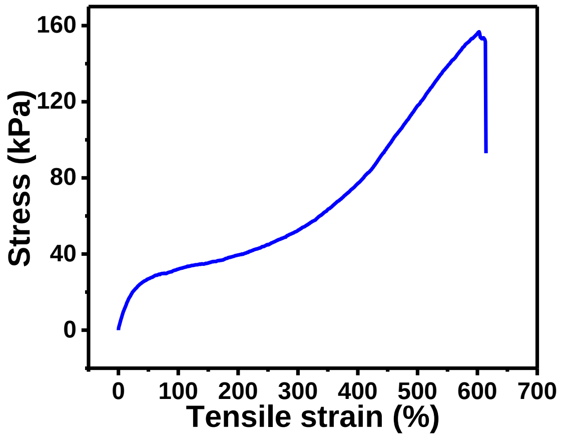

Supplementary Fig. 49. Tensile stress-strain curve of rGO/PPy/PUF1-UHP in *x* direction after heat treatment at 140 °C for 2 h. Source data are provided as a Source Data file.

Supplementary Tables

Supplementary Table 1. Starting compositions of the rGO/PUF-based aerogels (rGO/PPy/PUF1 and rGO/PPy/PUF2).

	Preparation of rGO/PUF aerogel		Deposition of PPy		
Reagent	GO (4 mg mL^{-1})	EDA (AR)	$FeCl_3$ (98%)	H_2O (DIW)	Pyrrole (99%)
Amount	4 mL	0.032 mL	0.2268 g	8 mL	0.066 mL

Supplementary Table 2. Starting compositions of the rGO/PU-based aerogel (rGO/PPy/PU).

	Preparation of rGO/PU aerogel				Deposition of PPy		
Reagent	GO (4 mg mL^{-1})	PU dispersion (50 wt% in water)	H_2O (DIW)	APTES (98%)	$FeCl_3$ (98%)	H_2O (DIW)	Pyrrole (99%)
Amount	8 mL	1.28 mL	8 mL	0.096 mL	0.2268 g	8 mL	0.066 mL

Supplementary Table 3. Starting compositions of the rGO/PVA/MF aerogel.

Reagent	GO (4 mg mL^{-1})	PVA (0.05 g mL^{-1})	EDA (AR)
Amount	4 mL	0.16 mL	0.032 mL

Supplementary Table 4. Percentage of element content by XPS surveys of different kinds of aerogels.

Sample	C 1s (%)	O 1s (%)	N 1s (%)	Si 2p (%)
APTES-crosslinked rGO/PU aerogel	72.44	21.04	4.25	2.28
APTES-crosslinked rGO aerogel	56.22	26.57	8.40	8.81
GO/PU aerogel without APTES	74.92	23.37	1.71	0

Supplementary Table 5. Stretchability of the reported typical stretchable aerogels.

Ref.	Published year	Composition	Elongation at break (%)	Reversible elongation (%)
1	2021	Poly(isocyanurate-urethane)	120	-
2	2020	Poly(caprolactone)	275	-
3	2021	Polyimide	25	-
4	2022	Cellulose	50	-
5	2020	Cellulose nanofiber/polyurethane(PU)	25	-
6	2018	MXene/polyimide	35	20
7	2022	Aramid nanofiber/carbon nanotube (CNT) /polypyrrole	26	-
8	2023	Reduced graphene oxide (rGO)/PU	100	-
9	2020	Graphene	20	-
10	2023	Graphene	420	400
11	2021	SiC-SiOx nanowire	22	20
12	2022	Aramid nanofiber composite	30	-
13	2023	Silica nanofiber	220	-
14	2022	Ceramic nanofiber	100	40
15	2022	Hypocrystalline ceramic nanofiber	40	-

Supplementary Table 6. Stretchability of the reported stretchable porous materials (mainly foams and sponges) other than aerogels.

Ref.	Published year	Composition	Elongation at break (%)	Reversible elongation (%)
16	2019	Poly(3,4-ethylenedioxythiophene): polystyrene sulfonate (PEDOT:PSS)	100	60
17	2015	Graphene/polyimide	16	-
18	2021	Carbon	80	60
19	2023	Si_3N_4 nanofiber	80	20
20	2021	Si_3N_4 Nanofiber	30	10
21	2017	Graphene/nickel particle	160	-
22	2014	CNT/graphene/ poly(dimethylsiloxane) (PDMS)	80	50
23	2017	CNT/PDMS	140	120
24	2015	Graphene/PDMS	-	77
25	2019	Graphene/modified silicone rubber	⩾100	40
26	2018	Graphene/PDMS	64	56
27	2020	Single-wall CNT/PDMS	62	50
28	2016	Metal/PDMS	-	50
29	2022	Graphene nanoplate/PDMS	~140	70
30	2018	CNT/polyurethane (PU)	320	300
31	2020	CNT/PU	350	~60
32	2020	CB/TPU	~600	~200
33	2020	Polystyrene/PU	70	40
34	2021	Fe_3O_4/PU	184	20
		CNT/ Fe_3O_4/PU	~90	20
35	2022	CNT/PU	~900	900
36	2020	CNT/PU foam	275	40
37	2018	rGO/AgNWs/PU	-	60
38	2021	Liquid metal/PUF	170	-
39	2020	GO/PU	~700	-
40	2015	TPU/graphene	~900	~400
41	2019	Liqiud metal/polydopamine-modified PU sponge	~150	50
42	2020	AgNW/TPU	-	~55
43	2020	PVDF-HFP foam with Ni particles	~240	−
44	2019	Polyacrylate-based polymer	650	−
45	2015	Graphene/PEDOT:PSS/PDMS	80	50
46	2021	Porous polyetheretherketone	235	-

		fibers		
47	2021	Polysulfone/PVDF composite fibrous sponge	~110	-
48	2021	PEMP/OEGDA polyHIPE fibers	155	-
49	2018	Graphene foam/PPy	-	50
50	2021	Wavy graphene foam/TPU	400	200
51	2014	Graphene/AgNW hybrid foam	54	40
52	2020	CNT-GO@melamine sponge	50	10
53	2019	Silicon sponge	50	25
54	2020	PEDOT:PSS/graphene composite sponge	-	50

Supplementary Notes

Supplementary Note 1. Density, porosity, and pore size of typical materials

As shown in Supplementary Fig. 12, the bulk densities of rGO/PPy/PUF1, rGO/PPy/PUF2, rGO/PVA/MF, and rGO/PPy/PU are 90, 40, 19, and 77 mg cm^{-3}, respectively, confirming the low densities of the pristine rGO/polymer composite aerogels. After hot pressing, the bulk densities of rGO/PPy/PUF1-UHP, rGO/PPy/PUF1-BHP, rGO/PPy/PUF1-THP, rGO/PPy/PUF2-UHP, rGO/PVA/MF-UHP, rGO/PPy/PU-UHP, rGO/PPy/PU-BHP, and rGO/PPy/PU-THP are 370, 370, 700, 280, 70, 330, 330, and 650 mg cm^{-3}, respectively.

The porosities of different materials are summarized in Supplementary Fig. 13. The porosities of rGO/PPy/PUF1, rGO/PPy/PUF2, rGO/PVA/MF, and rGO/PPy/PU reach 91%, 96%, 98%, and 92%, respectively. After hot pressing, the porosities of rGO/PPy/PUF1-UHP, rGO/PPy/PUF1-BHP, rGO/PPy/PUF1-THP, rGO/PPy/PUF2-UHP, rGO/PVA/MF-UHP, rGO/PPy/PU-UHP, rGO/PPy/PU-BHP, and rGO/PPy/PU-THP are 63%, 63%, 30%, 72%, 95%, 67%, 67%, and 35%, respectively. The porosities of all the uniaxially and biaxially hot-pressed materials are higher than 60%. In particular, the porosity of the hot-pressed rGO/PVA/MF-UHP is higher than 90%. As we can see, although the bulk density increases and porosity decreases after hot pressing, the resultant materials still possess porous structures.

As shown in the SEM images (Fig. 2), the foam- or honeycomb-like highly porous structures are converted into folded and reentrant porous structures with smaller pores after uniaxial, biaxial, and triaxial hot pressing. The pore sizes of rGO/PPy/PUF1 and rGO/PPy/PU are mainly in the range of 100-500 μm and 50-200 μm, respectively. By contrast, the pore sizes of rGO/PPy/PUF1-UHP, rGO/PPy/PUF1-BHP, and rGO/PPy/PUF1-THP are mainly in the range of 30-150 μm, 25-150 μm,

and 20-100 μm, respectively, while those of rGO/PPy/PU-UHP, rGO/PPy/PU-BHP, and rGO/PPy/PU-THP are mainly in the range of 20-150 μm, 20-130 μm, and 10-80 μm, respectively.

Supplementary Note 2. Analyses on chemical structures revealed by XRD patterns, Raman spectra, and FTIR spectra

As presented in the XRD patterns (Supplementary Fig. 17a-c), PUF1 shows a broad peak at approximately 19.5°, corresponding to the carbon chains with regular interplanar spacing in PUF1, while MF exhibits no obvious characteristic peak.[10] The rGO/polymer aerogels (rGO/PPy/PUF1, rGO/PPy/PU, and rGO/PVA/MF) present an obvious peak at approximately 21°, resulting from the synergistic effect of rGO and the polymers (Supplementary Fig. 17a-c). For the hot-pressed rGO/polymer elastomers, the intensity of this characteristic peak becomes higher, probably because rGO is further reduced during hot pressing.

The Raman spectra of the pristine rGO/polymer aerogels and hot-pressed elastomers exhibit the D band at approximately 1327 cm^{-1} and G band at approximately 1580 cm^{-1}, corresponding to the characteristic bands of rGO, confirming the successful incorporation of rGO in the composite materials (Supplementary Fig. 17d-f). [10] For rGO/PPy/PUF1, rGO/PPy/PUF1-UHP, rGO/PPy/PU, and rGO/PPy/PU-UHP, there are two new bands at approximately 957 and 1055 cm^{-1}, which correspond to the ring deformation vibration of PPy and the in-plane deformation of C-H and in-plane bending of N-H in PPy, respectively. [55]

As shown in the FTIR spectra (Supplementary Fig. 17g-i), the peaks of PUF1 and the rGO/PU aerogel at 2870, 1721, 1086, and 700 cm^{-1} correspond to the stretching vibration of C-H, C=O, C-O, and in-plane vibration of C-H, respectively.[8,10,56] These peaks are also observed but become weaker in rGO/PPy/PUF1, rGO/PPy/PUF1-UHP, rGO/PPy/PU, and rGO/PPy/PU-UHP because of the incorporation of rGO and PPy. In the case of MF, the broad peak at approximately 3325 cm^{-1} corresponds to the stretching vibration of N-H, which is not obvious for rGO/PVA/MF and rGO/PVA/MF-UHP because of the incorporation of PVA and rGO (Supplementary Fig. 16i). The peaks at approximately 1315 cm^{-1} and 808 cm^{-1} correspond to the bending vibration of C-H and triazine ring, respectively.[57] The peaks at 1533 and 1069 cm^{-1} correspond to the N-H and C-O-C groups, respectively. [58]

Supplementary Note 3. XPS analysis of typical aerogels

XPS spectrum of the APTES-crosslinked rGO/PU and rGO aerogels clearly shows five peaks at 532.1, 399.3, 284.3, 152.8 and 101.1 eV, corresponding to O 1*s*, N 1*s*, C 1*s*, Si 2*s*, and Si 2*p*, respectively

(Supplementary Figs. 18, 19).[59] For the GO/PU aerogel without APTES, there are three peaks at 532.0, 398.9, and 284.3 eV, corresponding to O 1*s*, N 1*s*, and C 1*s*, respectively (Supplementary Fig. 20), where the peak of N 1*s* is attributed to PU. There are no peaks corresponding to Si 2*s* and Si 2*p* in the GO/PU aerogel without APTES (Supplementary Fig. 20). The resultant percentage of element content of different aerogels is listed in Supplementary Table 4.

The O 1*s* spectrum of the APTES-crosslinked rGO/PU aerogel can be divided into three peaks at 532.7, 532.1, and 531.4 eV, representing C-O, Si-O, and C=O bonds, respectively (Supplementary Fig. 18).[60] The N 1*s* spectra of the APTES-crosslinked rGO/PU aerogel and GO/PU aerogel without APTES show only one peak at 398.9 eV, representing -NH- bond.[61] The N 1*s* spectrum of the APTES-crosslinked rGO aerogel shows two peaks at 401.2 and 398.9 eV, representing $-NH_2/NH_3^+$ and -NH- bonds, respectively. [61] The -NH- bond of the APTES-crosslinked rGO aerogel is supposed to be attributed to the nucleophilic displacement reaction between epoxy groups in GO and amino groups in APTES (Fig. 1b and Supplementary Fig. 2) [61, 62]. The -NH- bond of the APTES-crosslinked rGO/PU aerogel is supposed to come from the -COO-NH- groups of PU and the nucleophilic displacement reaction between epoxy groups in GO and amino groups in APTES. The C 1*s* spectra of the APTES-crosslinked rGO/PU aerogel can be divided into five peaks at 288.0, 286.1, 285.4, 284.1, and 283.5 eV, representing C=O, C-O, C-N, C-C, and C-Si bonds, respectively (Supplementary Fig. 18).[17,61, 63] For the APTES-crosslinked rGO/PU and rGO aerogels, the Si 2*p* spectrum can be divided into two peaks at 101.1 and 101.7 eV, corresponding to Si-C and Si-O-Si bonds, respectively (Supplementary Figs. 18, 19).[64] The Si-O-Si bond is attributed to the hydrolytic polycondensation of APTES. These results indicate that GO nanosheets can be crosslinked by APTES via a C-N coupling reaction between epoxy groups of GO and amino groups of APTES combined with the hydrolytic polycondensation of alkoxy groups of APTES. Besides, the gel forms more quickly after adding APTES in the GO dispersion, providing an indirect evidence of the crosslinks between APTES and GO nanosheets.

Supplementary Note 4. **Interfaces between the polymer and rGO, morphology and crystal size of rGO**

As shown in SEM and optical microscope images of pristine rGO/PPy/PUF aerogels and hot-pressed porous rGO/PPy/PUF elastomers (Supplementary Figs. 9, 10), the crosslinked rGO nanosheets are

well deposited on the surface of the PUF skeletons and some pore walls of PUF are connected by the rGO nanosheets. rGO nanosheets and PUF skeletons are well connected with each other at the interfaces between them. The sizes of the rGO nanosheets in rGO/PPy/PUF system are mainly in the range of 50-500 μm, while those of the rGO nanosheets in rGO/PVA/MF system are mainly in the range of 20-200 μm (Supplementary Fig. 9). In the case of rGO/PU system, the SEM images indicate the well combination of rGO and PU (Supplementary Fig. 9). It is found that the rGO/PU gel forms more quickly compared with rGO gel without PU, indicating that there may be physical crosslinks between rGO and PU, which can be achieved by the hydrogen bonds between -NH-COO- groups in PU and -COOH (or -OH) groups in GO.

As shown in the TEM images (Supplementary Fig. 21), the size of GO nanosheets in the GO dispersion is in the range of approximately 1 to 6 μm. In addition, the microstructures of rGO and the interface between rGO and polymers were further investigated by TEM images. As shown in the TEM images of rGO/PPy/PU-UHP (Supplementary Fig. 22), the thicknesses of the rGO nanosheets are in the range of approximately 55-250 nm, while their sizes are in the range of approximately 1-7 μm. The interface between rGO and polymer can be observed clearly by the TEM images (Supplementary Fig. 22b). As we can see, the polymer and rGO in rGO/PPy/PU-UHP are well combined at the interface.

There are many irregular spherical particles on the surface of the skeletons of the aerogels rGO/PPy/PUF1, rGO/PPy/PUF2, and rGO/PPy/PU, which are well preserved after hot pressing (Fig. 2i-p and Supplementary Fig. 10). These particles are supposed to be PPy polymers, which were deposited by in-situ oxidation polymerization of pyrrole. The interface between PPy particles and rGO (or PU) is clearly observed in SEM images of the samples (Fig. 2 and Supplementary Fig. 10). The PPy particles remain attached to the skeleton of rGO/PU even after repeated washing, squeezing, hot pressing, stretching, and compression. Additionally, the conductivity of rGO/PPy/PU-UHP remains nearly unchanged after repeated stretching and compression. This indicates that there may be physical crosslinks between PPy and rGO (or PU), which can be achieved by the hydrogen bonds between -NH- groups in PPy and -COOH (or -OH) groups in GO (or -NH-COO- groups in PU).

Supplementary Note 5. Analyses on thermal conductivities of elastomers with different tensile strains

The thermal conductivity (λ_{total}) of porous materials is mainly composed of thermal conductivities of solid (λ_s), gas (λ_g), and radiation (λ_r). λ_s is given by $\lambda_s = \lambda_{s,s}\rho v/(\rho_s v_s)$ (1), where $\lambda_{s,s}$ is thermal

conductivity of solid of the basic material, ρ is apparent density, v is phonon velocity, ρ_s is true density, and v_s is estimated by the vibration of phonon in the skeleton.[65] The apparent density of the hot-pressed rGO/polymer elastomer decreases with increasing tensile strain, which will result in lower λ_s. λ_g is given by $\lambda_g = \phi\lambda_{g,0}/[1+2\beta l p_0 (pD)^{-1}]$ (2), where ϕ is porosity, $\lambda_{g,0}$ is thermal conductivity contributed by free gas without convection, β is a constant, l is mean free path of gas molecules, p is pressure of gas, p_0 is pressure of reference gas, and D is mean pore diameter of porous materials.[56,66] The pore size and porosity of the hot-pressed rGO/polymer elastomer increase with increasing tensile strain, which will lead to higher λ_g. λ_r is given by $\lambda_r = 16\sigma n^2 T^3/[3\rho e(T)]$ (3), where σ is Stefen-Boltzmann constant, n is complex refractive index, T is absolute temperature, and $e(T)$ is specific extinction coefficient.[66] The decreased apparent density of the hot-pressed elastomer with a larger tensile strain will result in higher λ_r. The increased λ_g and λ_r probably contribute to the lower thermal insulation of the hot-pressed rGO/polymer elastomer with larger tensile strains.

Supplementary Note 6. Thermal stability of the elastomers

To investigate the thermal stability of rGO/PPy/PUF1-UHP and rGO/PPy/PU-UHP, the thermogravimetric (TG) and derivative thermogravimetric (DTG) curves were measured in air by a TG analyzer (Supplementary Fig. 47). rGO/PPy/PUF1-UHP shows a significant weight loss of approximately 75% in the temperature range of 200-400 °C, which is accompanied with an intense peak of DTG at 297.7 °C. In the case of rGO/PPy/PU-UHP, it shows a significant weight loss of approximately 76% in the temperature range of 300-400 °C, which is accompanied with an intense peak of DTG at 358.3 °C. The TG and DTG curves indicate that rGO/PPy/PUF1-UHP and rGO/PPy/PU-UHP are thermally stable up to approximately 200 and 300 °C, respectively.

To investigate the influence of heat treatment on mechanical properties of the hot-pressed elastomers, compression-decompression curves of rGO/PPy/PUF1-UHP and rGO/PPy/PU-UHP after heat treatment at different temperatures (50, 100, and 140 °C for 2 h) were tested (Supplementary Fig. 48). After heat treatment at 140 °C, the length of rGO/PPy/PUF1-UHP and rGO/PPy/PU-UHP increases 25% and 10%, respectively, along x direction. By contrast, after heat treatment at 100 °C, the length of rGO/PPy/PUF1-UHP and rGO/PPy/PU-UHP only shows a slight increase along x direction (5% and 2%, respectively). There is no change in size for both rGO/PPy/PUF1-UHP and rGO/PPy/PU-

UHP after heat treatment at 50 °C. The compressive stresses at 80% strain for rGO/PPy/PUF1-UHP and rGO/PPy/PU-UHP decrease after heat treatment at 140 °C because of the volume expansion, while their compressive stresses at 80% strain show no obvious change after heat treatment at 100 and 50 °C (Supplementary Fig. 48). Both rGO/PPy/PUF1-UHP and rGO/PPy/PU-UHP after heat treatment at different temperatures (50, 100, and 140 °C) maintain high elasticities (Supplementary Fig. 48).

In addition, the tensile stress-strain curve of rGO/PPy/PUF1-UHP was measured after heat treatment at 140 °C for 2 h (Supplementary Fig. 49). After heat treatment at 140 °C for 2 h, the elongation at break of rGO/PPy/PUF1-UHP in *x* direction decreases from 810% to 603%. The decreased elongation at break is probably attributed to the volume expansion after heat treatment.

Based on the above analysis on compressive and tensile stress-strain curves before and after heat treatment at different temperatures, it can be concluded that the mechanical properties of typical hot-pressed porous elastomers remain nearly unchanged after heat treatment at temperatures below 100 °C. Since the thermal insulation and management demonstrations were carried out at the temperature of 51 °C, both rGO/PPy/PUF1-UHP and rGO/PPy/PU-UHP are thermally stable at this applied condition. In addition, the storage moduli of rGO/PPy/PUF1 and rGO/PPy/PU show no obvious decrease in the temperature range of 25-55 °C (Supplementary Fig. 14), indicating that the mechanical properties of the materials are relatively stable at this applied condition.

Supplementary References

[1] Malakooti, S., Rostami, S., Churu, H. G., Luo, H., Clark, J., Casarez, F., Rettenmaier, O., Daryadel, S., Minary-Jolandan, M., Sotiriou-Leventis, C., Leventis, N. & Lu, H. Scalable, hydrophobic and highly-stretchable poly(isocyanurate–urethane) aerogels. *RSC Adv.* **8**, 21214-21223 (2018).

[2] Wang, C., Wang, M., Ying, S. & Gu, J. Fast chemo-responsive shape memory of stretchable polymer nanocomposite aerogels fabricated by one-step method. *Macomol. Mater. Eng.* **305**, 1900602 (2020).

[3] Li, X., Dong, G., Liu, Z. & Zhang, X. Polyimide aerogel fibers with superior flame resistance, strength, hydrophobicity, and flexibility made via a universal sol−gel confined transition strategy. *ACS Nano* **15**, 4759-4768 (2021).

[4] Li, Q., Yuan, Z., Zhang, C., Hu, S., Chen, Z., Wu, Y., Chen, P., Qi, H. & Ye, D. Tough, highly

oriented, super thermal insulating regenerated all-cellulose sponge-aerogel fibers integrating a graded aligned nanostructure. *Nano Lett.* **22**, 3516-3524 (2022).

[5] Wang, Y., Li, X., Cheng, H., Wang, B., Feng, X., Mao, Z. & Sui, X. Facile fabrication of robust and stretchable cellulose nanofibers/polyurethane hybrid aerogels. *ACS Sustainable Chem. Eng.* **8**, 8977-8985 (2020).

[6] Liu, J., Zhang, H. -B., Xie, X., Yang, R., Liu, Z., Liu, Y. & Yu, Z. -Z. Multifunctional, superelastic, and lightweight MXene/polyimide aerogels. *Small* **14**, 1802479 (2018).

[7] Huang, J., Li, J., Xu, X., Hua, L. & Lu, Z. In situ loading of polypyrrole onto aramid nanofiber and carbon nanotube aerogel fibers as physiology and motion sensors. *ACS Nano* **16**, 8161-8171 (2022).

[8] Zhang, X., Hu, Z., Sun, Q., Liang, X., Gu, P., Huang, J. & Zu, G. Bioinspired gradient stretchable aerogels for ultrabroad-range-response pressure-sensitive wearable electronics and high-efficient separators. *Angew. Chem. Int. Ed.* **62**, e202213952 (2023).

[9] Pang, K., Song, X., Xu, Z., Liu, X., Liu, Y., Zhong, L., Peng, Y., Wang, J., Zhou, J., Meng, F., Wang, J. & Gao, C. Hydroplastic foaming of graphene aerogels and artificially intelligent tactile sensors. *Sci. Adv.* **6**, eabd4045 (2020).

[10] Yang, G., Zhang, X., Wang, R., Liu, X., Zhang, J., Zong, L. & Yang, H. Ultra-stretchable graphene aerogels at ultralow temperatures. *Mater. Horiz.* **10**, 1865-1874 (2023).

[11] Su, L., Wang, H., Jia, S., Dai, S., Niu, M., Ren, J., Lu, X., Cai, Z., Lu, D., Li, M., Xu, L., Guo, S. -W., Zhuang, L. & Peng, K. Highly stretchable, crack-insensitive and compressible ceramic aerogel. *ACS Nano* **15**, 18354-18362 (2021).

[12] He, H., Wei, X., Yang, B., Liu, H., Sun, M., Li, Y., Yan, A., Tang, C. Y., Lin, Y. & Xu, L. Ultrastrong and multifunctional aerogels with hyperconnective network of composite polymeric nanofibers. *Nat. Commun.* **13**, 4242 (2022).

[13] Gao, F., Tong, Z., Xiao, W., Liu, Q., Lu, J., Hou, Y., He, Q., Gao, X., Cheng, D., Zhan, X., Ma, Y. & Zhang, Q. Structural engineering of hierarchical aerogels hybrid networks for efficient thermal comfort management and versatile protection. *Small* **19**, 2301164 (2023).

[14] Cheng, X., Liu, Y. –T., Si, Y., Yu, J. & Ding, B. Direct synthesis of highly stretchable ceramic nanofibrous aerogels via 3D reaction electrospinning. *Nat. Commun.* **13**, 2637 (2022).

[15] Guo, J., Fu, S., Deng, Y., Xu, X., Laima, S., Liu, D., Zhang, P., Zhou, J., Zhao, H., Yu, H., Dang,

S., Zhang, J., Zhao, Y., Li, H. & Duan, X. Hypocrystalline ceramic aerogels for thermal insulation at extreme conditions. *Nature* **606**, 909-916 (2022).

[16] Chen, G., Rastak, R., Wang, Y., Yan, H., Feig, V., Liu, Y., Jiang, Y., Chen, S., Lian, F., Molina-Lopez, F., Jin, L., Cui, K., Chung, J. W., Pop, E., Linder, C. & Bao, Z. Strain- and strain-rate-invariant conductance in a stretchable and compressible 3D conducting polymer foam. *Matter* **1**, 205-218 (2019).

[17] Qin, Y., Peng, Q., Ding, Y., Lin, Z., Wang, C., Li, Y., Xu, F., Li, J., Yuan, Y., He, X. & Li, Y. Lightweight, superelastic, and mechanically flexible graphene/polyimide nanocomposite foam for strain sensor application. *ACS Nano* **9**, 8933-8941 (2015).

[18] Gao, H. -L., Wang, Z. -Y., Cui, C., Bao, J. -Z., Zhu, Y. -B., Xia, J., Wen, S. -M., Wu, H. -A. & Yu, S. -H. A highly compressible and stretchable carbon spring for smart vibration and magnetism sensors. *Adv. Mater.* **33**, 2102724 (2021).

[19] Li, M., Xiao, L., Guo, P., Ni, H., Lu, D., Xu, L., Wang, L., Zhang, J., Su, L. & Wang, H. Resilient and antipuncturing Si_3N_4 nanofiber sponge. *Nano Lett.* **23**, 1289-1297 (2023).

[20] Li, M., Su, L., Wang, H., Wan, P., Guo, P., Cai, Z., Gao, H., Zhang, Z. & Lu D. Stretchable and compressible Si_3N_4 nanofiber sponge with aligned microstructure for highly efficient particulate matter filtration under high-velocity airflow. *Small* **17**, 2100556 (2021).

[21] Yang, C., Xu, Y., Man, P., Zhang, H., Huo, Y., Yang, C., Li, Z., Jiang, S. & Man, B. Formation of large-area stretchable 3D graphene–nickel particle foams and their sensor applications. *RSC Adv.* **7**, 35016-35026 (2017).

[22] Chen, M., Zhang, L., Duan, S., Jing, S., Jiang, H. & Li, C. Highly stretchable conductors integrated with a conductive carbon nanotube/graphene network and 3D porous poly(dimethylsiloxane). *Adv. Funct. Mater.* **24**, 7548-7556 (2014).

[23] Fan, Y. J., Meng, X. S., Li, H. Y., Kuang, S. Y., Zhang, L., Wu, Y., Wang, Z. L., Zhu, G. Stretchable porous carbon nanotube-elastomer hybrid nanocomposite for harvesting mechanical energy. *Adv. Mater.* **29**, 1603115 (2017).

[24] Jeong, Y. R., Park, H., Jin, S. W., Hong, S. Y., Lee, S. -S. & Ha, J. S. Highly stretchable and sensitive strain sensors using fragmentized graphene foam. *Adv. Funct. Mater.* **25**, 4228-4236 (2015).

[25] Xu, M., Li, F., Zhang, Z., Shen, T., Zhang, Q. & Qi, J. Stretchable and multifunctional strain

sensors based on 3D graphene foams for active and adaptive tactile imaging. *Sci. China Mater.* **62**, 555-565 (2019).

[26] Long, Y., Zhao, X., Jiang, X., Zhang, L., Zhang, H., Liu, Y. & Zhu, H. A porous graphene/polydimethylsiloxane composite by chemical foaming for simultaneous tensile and compressive strain sensing. *FlatChem* **10**, 1-7 (2018).

[27] Liang, J., Wang, S., Yu, H., Zhao, X., Wang, H., Tong, Y., Tang Q. & Liu, Y. Solution-processed PDMS/SWCNT porous electrodes with high mass loading: toward high performance all-stretchable-component lithium ion batteries. *Sustainable Energy Fuels* **4**, 2718-2726 (2020).

[28] Liang, S., Li, Y., Yang, J., Zhang, J., He, C., Liu, Y. & Zhou, X. 3D stretchable, compressible, and highly conductive metal-coated polydimethylsiloxane sponges. *Adv. Mater. Technol.* **1**, 1600117 (2016).

[29] Hu, H., Ma, Y., Yue, J. & Zhang, F. Porous GNP/PDMS composites with significantly reduced percolation threshold of conductive filler for stretchable strain sensors. *Compos. Commun.* **29**, 101033 (2022).

[30] Wang, X., Sun, H., Yue, X., Yu, Y., Zheng, G., Dai, K., Liu, C. & Shen, C. A highly stretchable carbon nanotubes/thermoplastic polyurethane fiber-shaped strain sensor with porous structure for human motion monitoring. *Compos. Sci. Technol.* **168**, 126-132 (2018).

[31] Fan, H., Li, Q., Li, K., Hou, C., Zhang, Q., Li, Y. & Wang, H. Stretchable electrothermochromic fibers based on hierarchical porous structures with electrically conductive dual-pathways. *Sci. China Mater.* **63**, 2582-2589 (2020).

[32] Yue, X., Jia, Y., Wang, X., Zhou, K., Zhai, W., Zheng, G., Dai, K., Mi, L., Liu, C. & Shen, C. Highly stretchable and durable fiber-shaped strain sensor with porous core-sheath structure for human motion monitoring. *Compos. Sci. Technol.* **189**, 108038 (2020).

[33] Wu, H., Li, Y., Zhao, L., Wang, S., Tian, Y., Si, Y., Yu, J. & Ding, B. Stretchable and superelastic fibrous sponges tailored by "stiff−soft" bicomponent electrospun fibers for warmth retention. *ACS Appl. Mater. Interfaces* **12**, 27562-27571 (2020).

[34] Zheng, J., Wei, X., Li, Y., Dong, W., Li, X., E, S., Wu, Z. & Wen, J. Stretchable polyurethane composite foam triboelectric nanogenerator with tunable microwave absorption properties at elevated temperature. *Nano Energy* **89**, 106397 (2021).

[35] Zhu, G., Li, H., Peng, M., Zhao, G., Chen, J. & Zhu, Y. Highly-stretchable porous thermoplastic polyurethane/carbon nanotubes composites as a multimodal sensor. *Carbon* **195**, 364-371 (2022).

[36] Huang, K., Chen, M., He, G., Hu, X., He, W., Zhou, X., Huang Y. & Liu, Z. Stretchable microwave absorbing and electromagnetic interference shielding foam with hierarchical buckling induced by

solvent swelling. *Carbon* **157**, 466–477 (2020).

[37] Luan, Y., Zhang, S., Nguyen, T. H., Yang, W. & Noh, J. S. Polyurethane sponges decorated with reduced graphene oxide and silver nanowires for highly stretchable gas sensors. *Sens. Actuators, B* **265**, 609-616 (2018).

[38] Cho, D., Bhuyan, P., Sin, D., Kim, H., Kim, E. & Park, S. Stretchable, soft, and variable stiffness elastomer foam with positive and negative piezoresistivity enabled by liquid metal inclusion. *Adv. Mater. Technol.* **7**, 2101092 (2022).

[39] Song, B., He, W., Wang, X., Zeng, X., Cheng, M., Wu, F., Moon, K. & Wong, C. P. Fabrication of stretchable and conductive polymer nanocomposites based on interconnected graphene aerogel. *Compos. Sci. Technol.* **200**, 108430 (2020).

[40] Chen, Y., Li, Y., Xu, D. & Zhai, W. Fabrication of stretchable, flexible conductive thermoplastic polyurethane/graphene composites via foaming. *RSC Adv.* **5**, 82034-82041 (2015).

[41] Huang, Y., Yu, B., Zhang, L., Ning, N., & Tian, M. Highly stretchable conductor by self-assembling and mechanical sintering of a 2D liquid metal on a 3D polydopamine-modified polyurethane sponge. *ACS Appl. Mater. Interfaces* **11**, 48321-48330 (2019).

[42] Zhou, W., Yao, S., Wang, H., Du, Q., Ma, Y. & Zhu, Y. Gas-permeable, ultrathin, stretchable epidermal electronics with porous electrodes. *ACS Nano* **14**, 5798-5805 (2020).

[43] Guo, H., Tan, Y. J., Chen, G., Wang, Z., Susanto, G. J., See, H. H., Yang, Z., Lim, Z. W., Yang, L. & Tee, B. C. K. Artificially innervated self-healing foams as synthetic piezo-impedance sensor skins. *Nat. Commun.* **11**, 5747 (2020).

[44] Hou, Y., Fang, G., Jiang, Y., Song, H., Zhang, Y. & Zhao, Q. Emulsion lyophilization as a facile pathway to fabricate stretchable polymer foams enabling multishape memory effect and clip application. *ACS Appl. Mater. Interfaces* **11**, 32423-32430 (2019).

[45] Chen, M., Duan, S., Zhang, L., Wang, Z. & Li, C. Three-dimensional porous stretchable and conductive polymer composites based on graphene networks grown by chemical vapour deposition and PEDOT: PSS coating. *Chem. Commun.* **51**, 3169-3172 (2015).

[46] Li, M., Tian, F., Jiang, J., Zhou, M., Chen, Q., Zhao, D. & Zhai, W. Robust and multifunctional porous polyetheretherketone fiber fabricated via a microextrusion CO_2 foaming. *Macromol. Rapid Commun.* **42**, 2100463 (2021).

[47] Feng, Y., Zong, D., Hou, Y., Yin, X., Zhang, S., Duan, L., Si, Y., Jia, Y. & Ding, B. Gradient structured micro/nanofibrous sponges with superior compressibility and stretchability for broadband sound absorption. *J. Colloid Interface Sci.* **593**, 59-66 (2021).

[48] Jiang, B., Zhang, T., Xu, Z. & Zhao, Y. Wet-spun porous fibers from high internal phase emulsions: Continuous preparation and high stretchability. *J. Polym. Sci.* **59**, 1055-1064 (2021).

[49] Ren, J., Ren, R. P. & Lv, Y. K. Stretchable all-solid-state supercapacitors based on highly conductive polypyrrole-coated graphene foam. *Chem. Eng. J.* **349**, 111-118 (2018).

[50] Wang, L., Wu, Y., Li, Z., Jiang, N. & Niu, K. Wavy graphene foam reinforced elastomeric composites for large-strain stretchable conductors. *Composites, Part B* **224**, 109179 (2021).

[51] Wu, C., Fang, L., Huang, X. & Jiang, P. Three-dimensional highly conductive graphene–silver nanowire hybrid foams for flexible and stretchable conductors. *ACS Appl. Mater. Interfaces*. **6**, 21026-21034 (2014).

[52] Liu, P. & Chen, W. Microwave-assisted selective heating to rapidly construct a nano-cracked hollow sponge for stretch sensing. *J. Mater. Chem. C* **8**, 9391-9400 (2020).

[53] Li, L., Hu, T., Yang, Y. & Zhang, J. Strong, compressible, bendable and stretchable silicone sponges by solvent-controlled hydrolysis and polycondensation of silanes. *J. Colloid Interface Sci.* **540**, 554-562 (2019).

[54] Yan, W., Li, J., Zhang, G., Wang, L. & Ho, D. A synergistic self-assembled 3D PEDOT: PSS/graphene composite sponge for stretchable microsupercapacitors. *J. Mater. Chem. A* **8**, 554-564 (2020).

[55] Yin, Y., Prabhakar, M., Ebbinghaus, P., da Silva, C. C. & Rohwerder, M. Neutral inhibitor molecules entrapped into polypyrrole network for corrosion protection. *Chem. Eng. J.* **440**, 135739 (2022).

[56] Zu, G., Shimizu, T., Kanamori, K., Zhu, Y., Maeno, A., Kaji, H., Shen, J. & Nakanishi K. Transparent, superflexible doubly cross-linked polyvinylpolymethylsiloxane aerogel superinsulators via ambient pressure drying. *ACS Nano* **12**, 521-532 (2018).

[57] Ji, C., Zhang, K., Li, L., Chen, X., Hu, J., Yan, D., Xiao, G. & He, X. High performance graphene-based foam fabricated by a facile approach for oil absorption. *J. Mater. Chem. A* **5**, 11263-11270 (2017).

[58] Yu, Y., Zhai, Y., Yun, Z., Zhai, W., Wang, X., Zheng, G., Yan, C., Dai, K., Liu, C. & Shen, C. Ultra-stretchable porous fiber-shaped strain sensor with exponential response in full sensing range and excellent anti-interference ability toward buckling, torsion, temperature, and humidity. *Adv. Electron. Mater.* **5**, 1900538 (2019).

[59] Wu, X., Fan, M., Shen, X., Cui, S. & Tan, G. Silica aerogels formed from soluble silicates and

methyl trimethoxysilane (MTMS) using CO_2 gas as a gelation agent. *Ceram. Int.* **44**, 821-829 (2018).

[60] Smith, M., Scudiero, L., Espinal, J. & McEwen, J. Improving the deconvolution and interpretation of XPS spectra from chars by ab initio calculations. *Carbon* **110**, 155-171 (2016).

[61] Hu, H., Zhao, Z., Wan, W., Gogotsi, Y. & Qiu, J. Ultralight and highly compressible graphene aerogels. *Adv. Mater.* **25**, 2219-2223 (2013).

[62] Zu, G., Kanamori, K., Nakanishi, K., Lu, X., Yu, K., Huang, J. & Sugimura, H. Superelastic multifunctional aminosilane-crosslinked graphene aerogels for high thermal insulation, three-component separation, and strain/pressure-sensing arrays. *ACS Appl. Mater. Interfaces* **11**, 43533-43542 (2019).

[63] Bashouti, M. Y, Paska, Y., Puniredd, S. R., Thomas, T., Christiansen, S. & Haick, H. Silicon nanowires terminated with methyl functionalities exhibit stronger Si–C bonds than equivalent 2D surfaces. *Phys. Chem. Chem. Phys.* **11**, 3845-3848 (2009).

[64] Gupta, P., Sathwane, M., Chhajed, M., Verma, C., Grohens, Y., Seantier, B., Agrawal, A. K & Maji, P. K. Surfactant assisted in situ synthesis of nanofibrillated cellulose/polymethylsilsesquioxane aerogel for tuning its thermal performance. *Macromol. Rapid Commun.* **44**, 2200628 (2023).

[65] Zu, G., Zeng, S., Yang, B. & Huang, J. Transparent, ultraflexible, and superinsulating nanofibrous biocomposite aerogels via ambient pressure drying. *J. Mater. Chem. A* **9**, 5769-5779 (2021).

[66] Lu, X., Arduini-Schuster, M. C., Kuhn, J., Nilsson, O., Fricke, J. & Pekala, R. W. Thermal conductivity of monolithic organic aerogels. *Science* **255**, 971-972 (1992).
