# Supplementary material for: Stretchable and negative-Poisson-ratio porous metamaterials
Source: Nat Commun. 2024 Jan 9;15:392. doi: 10.1038/s41467-024-44707-3 (PMC10776607; doi:10.1038/s41467-024-44707-3)
Supplement: Supplementary file 3 — Description of Additional Supplementary Files [file 41467_2024_44707_MOESM3_ESM.pdf]

Description of Additional Supplementary Files

File Name: Supplementary Movie 1
Description: A tensile test in *x* direction on rGO/PPy/PUF1-UHP, showing an elongation at break of 810%.

File Name: Supplementary Movie 2
Description: A tensile test in *x* direction on rGO/PPy/PUF2-UHP, showing an elongation at break of 1250%.

File Name: Supplementary Movie 3
Description: A stretching-releasing test in *x* direction on rGO/PPy/PUF1-UHP with 700% tensile strain.

File Name: Supplementary Movie 4
Description: A stretching-releasing test in *x* direction on rGO/PPy/PUF2-UHP with 800% tensile strain.

File Name: Supplementary Movie 5
Description: A tensile test in *x* direction on rGO/PPy/PUF1-BHP with 300% tensile strain.

File Name: Supplementary Movie 6
Description: A stretching-releasing test in *y* direction on rGO/PPy/PUF1-BHP with 300% tensile strain.

File Name: Supplementary Movie 7
Description: A tensile test in *y* direction on rGO/PPy/PUF1-THP, showing an elongation at break of 340%.

File Name: Supplementary Movie 8
Description: FEA simulation of the structure variation of the elastomer during uniaxial hot pressing.

File Name: Supplementary Movie 9
Description: FEA simulation of the structure variation of the elastomer during biaxial hot pressing.

File Name: Supplementary Movie 10
Description: Monitoring the large tensile strain (0-300%) of a chest developer during exercise by using the strain sensor based on rGO/PPy/PUF1-UHP.
